# A Peptide‐Conjugated Probe with Cleavage‐Induced Morphological Change for Treatment on Tumor Cell Membrane

**DOI:** 10.1002/advs.202207228

**Published:** 2023-02-15

**Authors:** Wei Zhang, Jing‐Jing Hu, Rui Liu, Jun Dai, Lizhen Yuan, Yiheng Liu, Bochao Chen, Mingxing Gong, Fan Xia, Xiaoding Lou

**Affiliations:** ^1^ State Key Laboratory of Biogeology and Environmental Geology Engineering Research Center of Nano‐Geomaterials of Ministry of Education Faculty of Materials Science and Chemistry China University of Geosciences Wuhan 430074 China; ^2^ Department of Obstetrics and Gynecology Tongji Hospital Tongji Medical College Huazhong University of Science and Technology Wuhan 430030 China

**Keywords:** cell membrane, nanofibers, peptide‐conjugated probe, self‐assemble, tumor metastasis

## Abstract

Despite the promising advancements of in situ forming nanoassembly for the inhibition of tumor growth and metastasis, the lack of sufficient triggering sites and hardly controlling the forming position restrict their further developments. Herein, a smart transformable peptide‐conjugated probe (DMFA) with enzyme cleavage‐induced morphological change is designed for treatment on the tumor cell membrane. Specifically, after self‐assembling into nanoparticles and anchoring on the cell membrane with sufficient interaction sites rapidly and stably, DMFA will be efficiently cleaved into *α*‐helix forming part (DP) and *β*‐sheet forming part (LFA) by overexpressed matrix metalloproteinase‐2. Thus, the promoted Ca^2+^ influx by DP‐induced cell membrane breakage and decreased Na^+^/K^+^‐ATPase activity by LFA‐assembled nanofibers wrapping the cells can inhibit PI3K‐Akt signaling pathway, leading to the inhibition of tumor cell growth and metastasis. This peptide‐conjugated probe undergoes in situ morphological transformation on the cell membrane, exhibiting great potential in tumor therapy.

## Graphical abstract

To achieve the effective inhibition of tumor growth and metastasis, a smart transformable peptide‐conjugated probe DMFA with matrix metalloproteinase‐2 cleavage‐induced morphological change is designed. By ensuring the sufficient interaction sites between the *α*‐helix and phospholipid bilayer and the precise formation of *β*‐sheet‐based nanofibers outside the cell membrane, tumor growth and metastasis could be efficiently prevented by this treatment method.

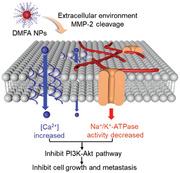

## 1 Introduction

As one of the major diseases, cancer still seriously endangers human life and health in the world due to the characteristics of uncontrollable growth and high invasion.^[^ ^1^ ^]^ When in the advanced stages of cancer, tumor cells will invade the surrounding tissues, escape from the primary tumor, then enter the blood and lymph circulation, and locate at other positions, like the lung and liver, of the body at the end.^[^ ^2^ ^]^ In fact, more than 90% of tumor‐associated mortality could be ascribed to metastasis because of its uncertainty, inoperability, and drug resistance.^[^ ^3^ ^]^ Therefore, effective tumor treatments not only need to eliminate the primary tumor but also could prevent the formation of tumor metastasis in the early stage.

After analyzing the process of tumor growth and metastasis, it is found that tumor cells secret some specific proteinases to degrade the extracellular matrix (ECM) that acts as physical scaffolding for embedded cells, thereby, realizing migration and invasion.^[^ ^4^ ^]^ Recently, utilizing biofunctional supramolecular nanofibers to construct artificial ECM and immobilize tumor cells has attracted great interest from researchers. For instance, taking advantage of the receptors on tumor cell membranes, conjugating the specific targeting unit to the assembled unit can realize the ligand‐receptor interaction‐induced reconstruction of nanofibers, thus achieving the inhibition of tumor growth and metastasis.^[^ ^5^ ^]^ While, the receptors on tumor cell membranes are dynamic and limited compared to the numerous proteins on membranes,^[^ ^6^ ^]^ leading to the relatively hard meeting and interaction between receptors and ligands. On the other hand, relying on external triggers like magnetism or photo, functionalized peptides or polymers can also realize the in situ transformation and form nanofibers.^[^ ^7^ ^]^ However, one unavoidably existent problem for the external triggers is that they cannot distinguish the position of nanofibers formation and may also induce the undesirable assembly in cells instead of outside the cells. Therefore, despite the advancements, it is necessary to propose a new strategy to ensure sufficient interaction sites and precise formation of nanofibers outside the tumor cells, and achieve effective inhibition of growth and metastasis.

**Figure 1:**
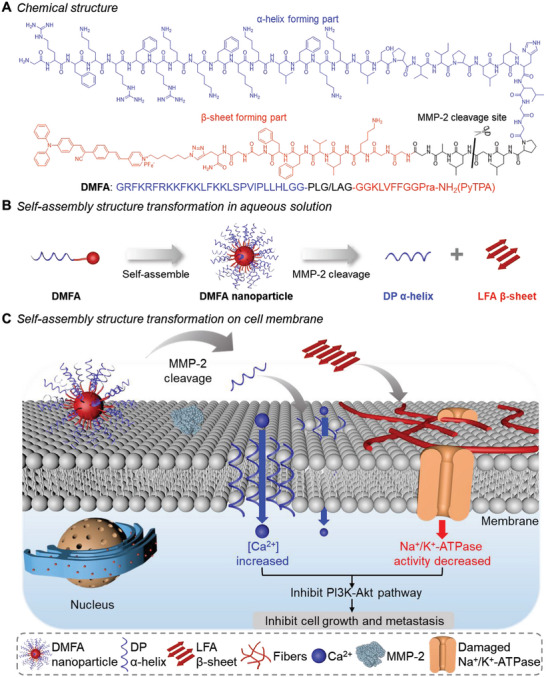
Schemes of peptide‐conjugated probe DMFA with cleavage‐induced morphological change on the cell membrane. A) Chemical structure of DMFA. B) DMFA self‐assembled into nanoparticles, which were cleaved by MMP‐2 and turned into *α*‐helix forming part (DP) and *β*‐sheet forming part (LFA). C) DMFA nanoparticle exhibited high affinity to the cell membrane and then was cleaved by pericellular MMP‐2. The released DP could damage the phospholipid bilayer structure and LFA could self‐assemble into nanofibers to wrap the tumor cells, further inhibiting the growth and metastasis of tumor cells.

Here, we designed a smart transformable peptide‐conjugated probe (DMFA) capable of undergoing morphological conversion cleaved by pericellular overexpressed matrix metalloproteinase‐2 (MMP‐2) to achieve high toxicity and metastasis inhibition on tumor cells. As shown in **Figure** **1**, DMFA contains three fragments: 1) GRFKRFRKKFKKLFKKLSPVIPLLHL, a positively charged amphiphilic peptide with *α*‐helix structure; 2) PLGLAG, an MMP‐2 responsive peptide substrate; 3) KLVFFGG(PyTPA), where KLVFF was a pentapeptide sequence derived from *β*‐amyloid (A*β*),^[^ ^8^ ^]^ and PyTPA was a fluorescence unit for image guidance.^[^ ^9^ ^]^ At first, DMFA could self‐assemble into nanoparticles with the exposure of the *α*‐helix peptide on the outside, thus, exhibiting high affinity to the cell membrane and achieving anchor rapidly and stably. After the pericellular MMP‐2 stimulation, DMFA could be cleaved into two parts efficiently, released *α*‐helix forming part (DP) and *β*‐sheet forming part (LFA). Further, through the in situ reconstruction, LFA could self‐assemble into the nanofibers on the tumor cell membrane, thereby, wrapping the tumor cells. Deep mechanism experiments demonstrated that the *α*‐helix structure of DP could damage the phospholipid bilayer structure and promote Ca^2+^ influx. Meanwhile, the LFA‐formed nanofibers could inhibit Na^+^/K^+^‐ATPase activity effectively, and combined with the above elevated intracellular Ca^2+^ concentration to affect the intracellular PI3K‐Akt signaling pathway, resulting in the inhibition of tumor growth and metastasis. In this study, by broadening the previous interaction between ligand and receptor to that between *α*‐helix and phospholipid bilayer, the interaction sites could be largely improved. Moreover, benefiting from the pericellular MMP‐2, the transformation location could be restricted and nanofibers would be assembled on the tumor cell membrane. This peptide‐based probe, which could undergo in situ morphological transformation on the cell membrane, exhibits great potential in tumor therapy.

## 2 Result and Discussion

### 2.1 Characteristics of DMFA and its Structural Transformation in Response to MMP‐2

**Figure 2:**
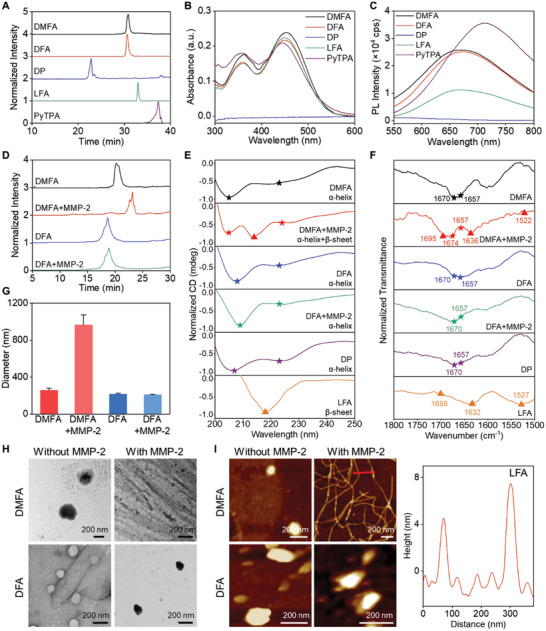
Characterization of different probes and their response to MMP‐2. A) HPLC spectra of different probes. B) UV–vis spectra and C) fluorescence spectra of different probes (10 µm). D) HPLC spectra of DMFA and DFA incubated with or without MMP‐2. E) CD spectra of DMFA, DFA, DP, LFA, DMFA after MMP‐2 incubation, and DFA after MMP‐2 incubation. The concentration of the probes was 20 µm. F) FTIR spectra, G) DLS (*n* = 3), H) TEM images, and I) AFM images of DMFA, DFA, DMFA after MMP‐2 incubation, and DFA after MMP‐2 incubation. The concentration of the probes was 10 µm. Inset panels show the height of the selected nanofibers. Characteristic peaks of *α*‐helix are marked as stars, while characteristic peaks of *β*‐sheet are marked as triangles in (E,F). Data were presented as mean ± SD.

To synthesize DMFA, PyTPA with azide group was first prepared based on our previous work, and the synthetic route is shown in Scheme S1, Supporting Information.^[^ ^9a^ ^]^ Meanwhile, the designed DMF peptide sequence mainly containing *α*‐helix forming peptide, MMP‐2 response peptide, as well as *β*‐sheet forming peptide, was prepared via solid‐phase synthesis. Then, PyTPA was conjugated at the C‐terminal position of alkynyl‐functionalized peptide DMF through a click reaction to obtain the transformable peptide‐conjugated probe DMFA (Scheme S2, Supporting Information).^[^ ^10^ ^]^ To better investigate the different functions of each part in DMFA, three control probes were designed and synthesized in similar methods including DFA (without MMP‐2 response part), DP (without *β*‐sheet forming part), and LFA (without *α*‐helix forming part) (Schemes S3 and S4 and Table S1, Supporting Information). All the above probes were purified by high‐performance liquid chromatography (HPLC) and their successful synthesis was proved by high‐resolution mass spectrometry (HRMS) (**Figure** **2**A and Figures S1–S5, Supporting Information). The UV–vis absorption spectra of DMFA, DFA, and LFA were similar to PyTPA with a maximum absorption peak at about 450 nm, proving the successful conjugation of PyTPA to the peptides (Figure 2B). DMFA, DFA, and LFA exhibited broad emission spectra from 600 to 800 nm with emission peaks located around 680 nm, which would be beneficial to confocal imaging (Figure 2C).

Subsequently, the responsiveness ability of DMFA to MMP‐2 was studied. As shown in Figure 2D, the HPLC peak of DMFA was at 20 min. After DMFA was incubated with MMP‐2, the original HPLC peak at 20 min disappeared and a new HPLC peak appeared at 23 min, which was proved to be LFA residue via HRMS analysis (Figure S6, Supporting Information), demonstrating that due to the presence of MMP‐2 recognition sequence PLGLAG, DMFA could be cleaved into DP and LFA as expected. As to DFA without MMP‐2 response sequence, there was no change after being incubated with MMP‐2 via both HPLC and HRMS (Figure S7, Supporting Information). By calculating the peak area in the HPLC spectrum, the efficiency of DMFA cleaved by MMP‐2 was more than 85% within 2 h (Figure S8, Supporting Information).

To further analyze the change of the secondary structure for DMFA after being cleaved by MMP‐2, circular dichroism (CD) was utilized. As shown in Figure 2E, a typical CD signal with two negative peaks at 207 and 223 nm of DP confirmed its *α*‐helix structure. The negative signal at 218 nm illustrated the *β*‐sheet structure of LFA. As to DMFA, it showed a typical *α*‐helix CD signal initially, while, after incubation with MMP‐2, the CD signals of *α*‐helix and *β*‐sheet structure were detected ascribing to the formation of DP and LFA.^[^ ^11^ ^]^ In contrast, without MMP‐2 responsive sequence, the *α*‐helix structure of DFA did not change before and after incubation with MMP‐2. Fourier transform infrared spectroscopy (FTIR) further illustrated the structural transformation of DMFA after MMP‐2 incubation (Figure 2F). The new peaks at 1522, 1636, and 1695 cm^−1^ in FTIR suggested the antiparallel *β*‐sheet structure generation after DMFA was incubated with MMP‐2.^[^ ^12^ ^]^ Next, we studied the structure formation of LFA and LF (Table S1, Supporting Information). Time gradient FTIR showed that LFA was easier to form antiparallel *β*‐sheet structures than LF, indicating that PyTPA was beneficial to the formation of antiparallel *β*‐sheet structures (Figures S9 and S10, Supporting Information).

Then, the self‐assembly morphological transitions of DMFA after incubation with MMP‐2 were examined. Due to the amphiphilic structure, DMFA itself could self‐assemble into spherical nanoparticles (Figure 2H) and the critical micelle concentration (CMC) assay illustrated that the lowest concentration for forming a self‐assembled structure was 2.69 µm (Figure S11, Supporting Information). Dynamic light scattering (DLS) demonstrated that the hydrodynamic diameter and zeta potential of DMFA nanoparticles was about 255 nm and +22.9 mV (Figure 2G and Figures S12 and S13, Supporting Information). While, after incubation with MMP‐2 for 2 h, the morphology of DMFA could be changed from nanoparticles to nanofibers with a width of 7.3 nm due to the generation of LFA (Figure 2H and Figure S14, Supporting Information) and along with the size increased to 960 nm (Figure 2G). Atomic force microscopy (AFM) images further showed that DMFA changed from nanoparticles to nanofibers after MMP‐2 treatment, and the minimal and maximal heights of nanofibers were 4.5 and 7.5 nm, respectively (Figure 2I and Figure S15, Supporting Information). Since DFA had no MMP‐2 response part, no obvious transformation was observed after MMP‐2 incubation. These results demonstrated that DMFA could be cleaved by MMP‐2 and then formed DP with *α*‐helix structure and LFA with antiparallel *β*‐sheet structure, thus achieving the morphology transition from nanoparticles to nanofibers.

### 2.2 Responsive Ability and Morphological Characterization on Cell Surface

**Figure 3:**
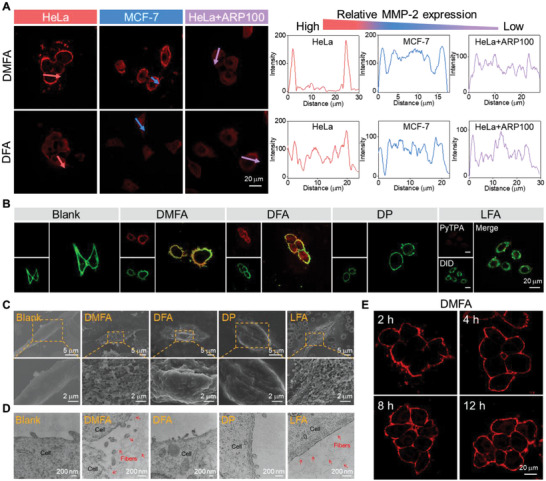
The responsive ability of DMFA to MMP‐2 in vitro. A) CLSM images and intensity of HeLa cells, MCF‐7 cells, and HeLa+ARP100 incubated with DMFA, DFA, and LFA for 4 h. The linear region across the HeLa cells, MCF‐7 cells, and HeLa cells+ARP100 are marked by red arrows, blue arrows, and purple arrows respectively. B) CLSM images of HeLa cells incubated with different probes for 4 h and then incubated with DID for 15 min. C) CLSM images of HeLa cells incubated with DMFA for 2, 4, 8, and 12 h. The concentration of DMFA was 20 µm. D) SEM images of HeLa cells incubated with different probes for 4 h. E) TEM images of HeLa cells incubated with different probes for 4 h. The concentration of the probes was 20 µm.

Encouraged by the satisfying MMP‐2 responsive ability of DMFA in solution, cellular experiments were further conducted to study the MMP‐2‐triggered transformation of DMFA nanoparticles on the cell membrane. After querying the database and measuring the MMP‐2 expression levels (Figures S16 and S17, Supporting Information), HeLa cells with overexpressed MMP‐2 in the ECM were selected as experimental cells. Besides, MCF‐7 cells or HeLa cells preincubated with MMP‐2 inhibitor ARP100 were also used as cells with medial or low MMP‐2 expression.^[^ ^13^ ^]^ As shown in **Figure** **3**A and Figures S18–S20, Supporting Information, after incubation with DMFA, the red fluorescence could be observed on the tumor cell membrane only in the HeLa cells with high MMP‐2 expression. When the MMP‐2 expression decreased, the red fluorescence began to appear in the cytoplasm, suggesting that with pericellular MMP‐2 cleavage, DMFA nanoparticles could transform into nanofibers to retain on the tumor cell membrane, if not they would be uptaken by the cells. The lysates of HeLa cells incubated with DMFA confirmed the generation of LFA by HRMS (Figure S21, Supporting Information). While in DFA‐incubated cells, no matter what the level of MMP‐2 expression was, obvious red fluorescence was observed in the cytoplasm, indicating that the DFA nanoparticles without MMP‐2 triggered morphology transformation could not retain on the cell membrane. As to LFA that could form nanofibers, the red fluorescence was on the cell membrane of these three kinds of cells regardless of the MMP‐2 levels. The above results illustrated that DMFA could be initially cut by the extracellular MMP‐2 and then formed LFA nanofibers.

After that, HeLa cells were incubated with different probes (red fluorescence channel) and then co‐incubated with the membrane staining dye DID (green fluorescence channel). The good overlap of red fluorescence and green fluorescence on the cell membrane in the DMFA and LFA group suggested that DMFA could form LFA nanofibers to stay on the cell membrane and avoid being uptaken by HeLa cells after being cleaved by MMP‐2 (Figure 3B and Figure S22, Supporting Information). 3D confocal laser scanning microscopy (CLSM) images also showed that DMFA could wrap the cell membrane of HeLa cells (Figure S23, Supporting Information).

Scanning electron microscopy (SEM) was then used to directly observe the nanofibers on the cell membrane (Figure 3C). After HeLa cells were incubated with different probes for 4 h, compared with the smooth membrane of HeLa cells without any treatments (blank group), a fibrillar network on the surface of HeLa cells incubated with DMFA was displayed, and similar fibrous structures were also observed in LFA group, which proved that the nanofibers could be formed by DMFA and wrap the cell membrane. Transmission electron microscopy (TEM) images showed similar results, there were abundant nanofibers with a width of 7.5 nm on the membrane of HeLa cells after incubation with DMFA for 4 h (Figure 3D). In order to simulate the tumor tissue, multicellular tumor spheroids (MCTs) were prepared and incubated with the probes, the CLSM images and 3D CLSM images illustrated that DMFA could also wrap the surfaces of MCTs (Figures S24 and S25, Supporting Information).

Real‐time CLSM images showed the gradual enrichment of DMFA on the cell membrane during 4 h incubation (Figure S26, Supporting Information). Further extending the incubation time to 12 h, the red fluorescence still remained on the surface of HeLa cells, suggesting the long‐term retention of DMFA on the cell membrane due to the formation of abundant nanofibers (Figure 3E and Figures S27–S30, Supporting Information). These data showed that DMFA could be cut by MMP‐2 and then self‐assembled nanofibers to wrap the cell membrane to avoid being internalized by HeLa cells.

### 2.3 *α*‐Helix and *β*‐Sheet Formation on Cell Membrane

**Figure 4:**
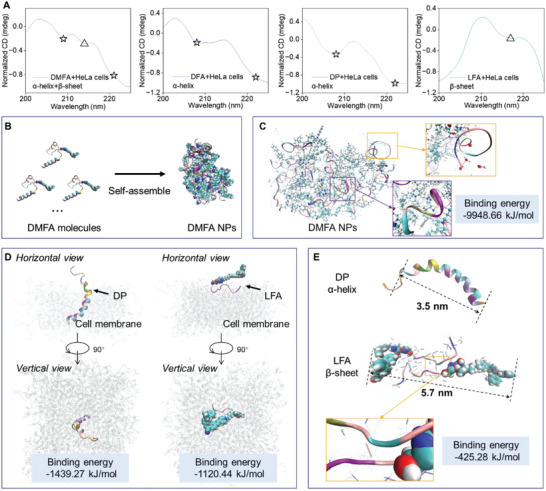
DMFA generated *α*‐helix and *β*‐sheet in vitro and computational simulation of the binding system and self‐assembly structure. A) CD of HeLa cell lysates after different treatments. B) Computational simulation of 20 DMFA single molecules self‐assembled into a nanoparticle. C) The structural simulation of the simplified model of DMFA nanoparticle. The enlarged figures represent the hydrogen bonds between the *α*‐helix part and the H_2_O molecule (yellow box) and the hydrogen bonds between amino acids (purple box), respectively. The size of the periodic box was 10 nm × 10 nm × 10 nm. D) The binding system computational simulation of DP and LFA to the cell membrane. E) Computational simulation of DP single molecule, and the binding energy of LFA single molecule self‐assembled into LFA anti‐parallel *β*‐sheet structure and the structural simulation of the simplified model of LFA anti‐parallel *β*‐sheet structure. The enlarged figure represents the intermolecular hydrogen bonds between amino acids. The size of the periodic box was 10 nm × 10 nm × 10 nm.

In the following, the HeLa cells after incubation with different probes for 24 h were lysed and tested by CD. As shown in **Figure** **4**A, there were three negative peaks at 209, 214, and 221 nm, confirming the existence of *α*‐helix structure and *β*‐sheet structure in the DMFA group ascribing to the formation of DP and LFA after cleavage. In contrast, without the MMP‐2 response sequence, only two negative peaks of the *α*‐helix structure CD signal at 208 and 222 nm were detected in the DFA group.

Then, the interaction between the probes and cell membrane at the single molecule level and the self‐assembly structure of probes were studied by computational simulation. As shown in Figure 4B,C, DMFA molecules exhibited obvious *α*‐helix structure. Due to the intramolecular hydrogen bonds, DMFA could self‐assemble into stable nanoparticles with a binding energy of −9948.66 kJ mol^−1^. Besides, from the assembly structure, DMFA could expose the *α*‐helix part on the outside with the observable hydrogen bonds to H_2_O molecules. To observe whether the *α*‐helix structure exposed on the outside was conducive to the combination of DMFA and cell membrane, DMFA, DFA, DP‐DEAC (DP labeled with DEAC fluorescent molecules, Figures S31 and S32, Supporting Information), and LFA were incubated with HeLa cells for a short time. The fluorescence signals could be observed on the cell membrane of the DMFA group, DFA group, and DP‐DEAC group after incubation for only 5 min, while no obvious fluorescence was observed on the cell membrane of the LFA group (Figure S33, Supporting Information), indicating that DMFA with *α*‐helix structure could rapidly anchor on the cell membrane. By simulating the binding system of DMFA to the cell membrane (Figure S34, Supporting Information), it was found that the binding energy of DMFA to the cell membrane was −3303.11 kJ mol^−1^, which meant that DMFA could stably bind to the cell membrane. In addition, the C‐terminal (including MMP‐2 response sequence) of DMFA located outside the cell membrane, which was conducive to the cleavage of DMFA by extracellular MMP‐2 and further formed DP and LFA. After DMFA was cut into DP and LFA, DP could still insert into the cell membrane, which helped DP destroy the membrane structure, and LFA could remain outside the cell membrane to better self‐assemble into fibers to wrap the cell membrane (Figure 4D). Moreover, the binding energies of DP and LFA to the membrane were respectively −1439.27 and −1120.44 kJ mol^−1^. Figure 4E shows that DP had a helical structure with a length of 4.4 nm. In addition, LFA had a linear structure and could stably self‐assemble into an antiparallel structure through intermolecular hydrogen bonds with a width of 5.7 nm and a binging energy of −425.28 kJ mol^−1^, which helped LFA further form nanofibers on the cell membrane. In contrast, the binding energy of LF (without PyTPA) self‐assembled into LF anti‐parallel structure was only −75.61 kJ mol^−1^, which reconfirmed that PyTPA contributed to the formation of anti‐parallel structure (Figure S35, Supporting Information). Combined with the above experimental data, it showed that DMFA could rapidly and stably anchor on the cell membrane, and then transformed into DP and LFA after being cut by MMP‐2. DP could insert into the cell membrane and destroy the cell membrane structure, and LFA could remain outside of the cell membrane and self‐assemble into fibers to wrap the cell membrane.

### 2.4 Inhibitory Effect of DMFA on HeLa Cells

**Figure 5:**
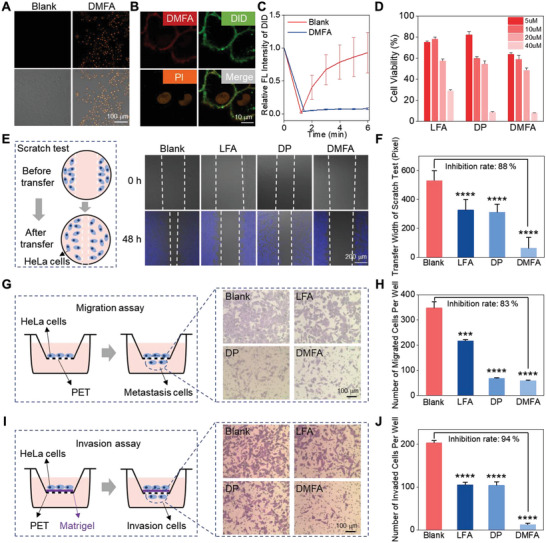
Inhibitory effect of DMFA on the growth and metastasis of HeLa cells in vitro. A) CLSM images of HeLa cells treated with different probes for 4 h and then incubated with PI for 20 min. B) CLSM images of HeLa cells incubated with DMFA (4 h), PI (20 min), and DID (15 min) in turn. C) The fluorescence recovery of DID which was incubated with HeLa cells pretreated with DMFA (*n* = 3). D) Cell viability of HeLa cells incubated with different probes for 24 h (*n* = 3). E) Schematic diagram of the scratch test, CLSM images, and F) quantitative analysis of HeLa cells incubated with different probes for 48 h and then incubated with Hoechst 33258 for 15 min. G) Schematic diagram of migration assay, transwell migration microscopy images, and H) quantitative analysis of HeLa cells incubated with different probes for 24 h (*n* = 3). I) Schematic diagram of invasion assay, transwell invasion microscopy images, and J) quantitative analysis of HeLa cells incubated with different probes for 24 h (*n* = 3). The concentration of the probes was 20 µm. Data were presented as mean ± SD. ***p < 0.001, ****p < 0.0001 versus corresponding blank group by Student's *t*‐test.

As the protective layer of the cell at the outermost, integrity and fluidity are two important parameters to evaluate the status of the cell membrane. First, the orange fluorescent signal of the propidium iodide (PI) indicator demonstrated that the DMFA was able to destroy the cell membrane integrity after incubation for 4 h (**Figure** **5**A and Figure S36, Supporting Information) since PI can enter into the cells through the broken cell membrane. The co‐incubation experiments of probes, PI, DID, and HeLa cells further showed that the integrity of cells was destroyed, and DMFA could retain on the cell membrane (Figure 5B and Figure S37, Supporting Information). Then, the fluidity of the cell membrane was tested. As shown in Figure 5C and Figure S38, Supporting Information, after the cell membrane was photobleached by laser irradiation, the membrane of cells treated with DMFA could not recover, illustrating that the fluidity of the cell membrane was damaged by DMFA. After verifying that the cell membrane was destroyed, cell cytotoxicity caused by probes was evaluated by 3‐(4,5‐Dimethylthiazol‐2‐yl)‐2,5‐diphenyl‐tetrazolium bromide (MTT) assay (Figure 5D and Figure S39, Supporting Information). All probes could produce cytotoxicity in a dose‐dependent manner, in which DMFA could produce a 37% cell growth inhibition rate at 5 µm and 93% cell growth inhibition rate at 40 µm, indicating that DMFA had high toxicity to HeLa cells.

In view of the above‐mentioned damaging effect of DMFA on the cell membrane, the inhibition effect of DMFA on metastasis and invasion of HeLa cells was then examined. First, a scratch test was carried out to study the inhibition of DMFA on the lateral transfer ability of HeLa cells. By comparing the position of the scratch edge at 0, 24, and 48 h (Figure 5E and Figure S40, Supporting Information), HeLa cells of the DP and LFA group transferred less than that in the blank group, while almost no cell transfer was observed in DMFA group, which suggested that DMFA could effectively inhibit the lateral transfer of HeLa cells. By calculating the average width of scratches (Figure 5F), it could be clearly understood that DMFA had the best cells transfer inhibition rate of about 88% in different experimental groups.

After that, transwell migration assays were used to evaluate the longitudinal migration ability of HeLa cells after incubation with DMFA, DP, and LFA. HeLa cells that migrated through the PET were stained with crystal violet and presented purple under the microscope (Figure 5G,H). Obviously, the cells of the blank group showed a high migration ability by observing a large number of cells, which was defined as a 100% cell migration rate. Treatment with LFA reduced the number of cells passing through PET, and the cell migration rate was about 62%. In contrast, DP and DMFA treatment significantly reduced the number of cells passing through PET, which decreased the cell migration rate to 20% and 17%, respectively, indicating that DP had a stronger ability to inhibit the longitudinal migration of HeLa cells than LFA, and the combination of these two parts (DMFA) showed the best ability to inhibit the longitudinal migration of HeLa cells.

To simulate the invasion of HeLa cells through the ECM in vivo, transwell invasion assays were carried out. As shown in Figure 5I,J, HeLa cells that passed through the PET coated with Matrigel (BD Biosciences) were stained with crystal violet and presented purple under the microscope. Compared with the blank group with a lot of invasive cells, which was defined as the 100% cell migration rate, the number of invasive cells of the DP and LFA group decreased, and the cell invasion rate of the DP and LFA group was 51% and 52%, respectively. However, after treatment with DMFA, few invasive cells were observed, and the cell invasion of DMFA treatment rate was reduced to 6%. These dates meant that DP and LFA had similar abilities to inhibit cell invasion, and DMFA had the best inhibition effect on cell invasion. As to DFA without an MMP‐2 response sequence, all three experiments illustrated that since it could not generate nanofibers, its inhibitory ability was still far weaker than DMFA (Figures S41 and S42, Supporting Information). The above results demonstrated that ascribing to the combination of *α*‐helix and *β*‐sheet, DMFA could effectively inhibit metastasis and invasion of HeLa cells.

### 2.5 Mechanism of DMFA Therapeutic Effect

**Figure 6:**
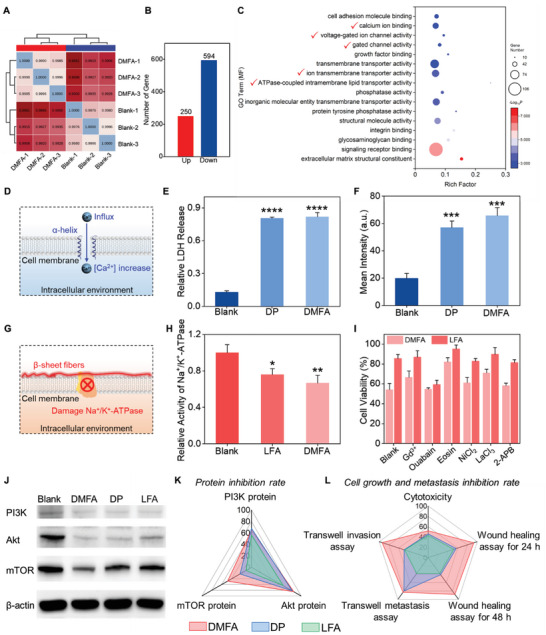
Mechanism of DMFA inhibiting growth and metastasis of HeLa cells. A) Repeated correlation studies of samples of HeLa cells incubated with or without DMFA (*n* = 3 per group). B) Genetic differences of HeLa cells incubated with or without DMFA. C) GO MF term enrichment analysis of differentially expressed genes between HeLa cells treated with or without DMFA. D) Schematic diagram of *α*‐helix destroying cell membrane and further causing Ca^2+^ influx. E) Relative LDH release assay of HeLa cells incubated with different probes (20 µm) for 24 h (*n* = 3). F) Mean intensity of CLSM images of HeLa cells treated with different probes (10 µm) for 30 min and then incubated with Ca^2+^ indicator for 30 min (*n* = 3). G) Schematic diagram of *β*‐sheet fibers wrapping cell membrane and further damaging Na^+^/K^+^‐ATPase. H) Relative activity of Na^+^/K^+^‐ATPase of HeLa cells incubated with different probes (20 µm) for 4 h (*n* = 3). I) Cell viability of HeLa cells pretreated with different inhibitors for 30 min and then DMFA or LFA (20 µm) was added for 4 h (*n* = 3). J) Western blotting analyses of the protein PI3K, Akt, and mTOR of HeLa cells incubated with different probes (20 µm) for 24 h. Radar plots of inhibition rates of different probes on K) PI3K‐Akt signaling pathway and L) HeLa cell's growth and metastasis. Data were presented as mean ± SD. *p < 0.05, **p < 0.01, ***p < 0.001, ****p < 0.0001 versus corresponding blank group by Student's *t*‐test.

To make it clear why DMFA could inhibit the growth and metastasis of HeLa cells, the transcriptomic changes of HeLa cells were analyzed after incubation with DMFA for 24 h. The gene expression indicated a remarkable difference in gene expression between DMFA and the blank group (**Figure** **6**A and Figure S43A, Supporting Information). It was found that 844 genes were differently expressed between DMFA and the blank group including 250 up‐regulated genes and 594 down‐regulated genes (Figure 6B and Figure S43B,C, Supporting Information). Gene ontology annotation classification statistics (including molecular function [GO MF], biological process [GO BP], and cellular component [GO CC]) showed that DMFA could affect ion‐, voltage‐gated ion channel‐, signal‐, membrane‐, migration‐, and so on (Figure 6C and Figure S43D,E, Supporting Information). Combing the above terms of Gene ontology and literatures, it could be hypothesized that DMFA could affect intracellular Ca^2+^ concentration and the activity of Na^+^/K^+^‐ATPase (membrane protein) by forming DP and LFA.^[^ ^14^, ^15^ ^]^ First, we examined whether the cell membrane damaged by *α*‐helix could affect intracellular Ca^2+^ concentration (Figure 6D), and a lactate dehydrogenase (LDH) release assay was conducted.^[^ ^14^ ^]^ As shown in Figure 6E, when the cell membrane was damaged by DP or DMFA with *α*‐helix secondary structure, the LDH was released from cells into the culture medium 6.19 times or 6.30 times more than that in the blank group, respectively. Then, the calcium ion probe (Fura‐2 AM) showed that the intracellular Ca^2+^ concentration of DP and DMFA group obviously increased compared with the blank group (Figure 6F and Figure S44, Supporting Information). These data suggested that the *α*‐helix structure formed after DMFA cleavage disrupted cell membranes and led to Ca^2+^ influx. Then, whether the *β*‐sheet structure formed by KLVFF could affect Na^+^/K^+^‐ATPase activity on the cell membrane was tested (Figure 6G).^[^ ^15^ ^]^ The data detected by the kit showed that LFA and DMFA with *β*‐sheet structure could reduce the Na^+^/K^+^‐ATPase activity of HeLa cells (Figure 6H). Furthermore, HeLa cells were pre‐treated with several bioactive Na^+^/K^+^‐ATPase inhibitors for 30 min including Gd^3+^ (stretch‐activated cation channel blocker), ouabain (binds to the cation‐binding site), eosin (binds to ATP‐binding site), NiCl_2_ (the T‐type voltage‐dependent channel blocker), LaCl_3_, and 2‐APB (non‐voltage‐sensitive channel blockers).^[^ ^16^ ^]^ As shown in Figure 6I, three inhibitors, including Gd^3+^, Eosin, and LaCl_3_, could reduce cell death after incubation with DMFA from 46% to 33%, 18%, and 29%, respectively, and reduce cell death after incubation with LFA from 15% to 13%, 5%, and 10%, respectively, which further illustrated that *β*‐sheet structure formed after DMFA cleavage could disrupt stretch‐activated cation channel, ATP‐binding site, and non‐voltage‐sensitive channel of Na^+^/K^+^‐ATPase.

Moreover, the Kyoto Encyclopedia of Genes and Genomes (KEGG) annotation classification showed that the differential genes enrichment of the PI3K‐Akt signaling pathway was significant (Figure S43F, Supporting Information), which suggested that the PI3K‐Akt signaling pathway of HeLa cells after incubation with DMFA was influenced, thus leading to the inhibition on growth and metastasis of HeLa cells.^[^ ^17^ ^]^ Since the PI3K‐Akt signaling pathway was related to the intracellular Ca^2+^ concentration and the activity of Na^+^/K^+^‐ATPase,^[^ ^18^ ^]^ the downregulation of protein PI3K, Akt, and mTOR (three key proteins in PI3K‐Akt signaling pathway) expression levels were evaluated by western blot assay (Figure 6J), illustrating that DMFA, DP, and LFA could inhibit PI3K‐Akt signaling pathway. These data suggested that after DMFA converted to DP and LFA, DP could damage the cell membrane and lead to the increase of intracellular Ca^2+^, and LFA could wrap the cell membrane and disrupt Na^+^/K^+^‐ATPase, both of which would inhibit intracellular PI3K‐Akt signaling pathway. By summarizing the above data, we obtained the inhibition rates of different probes on the PI3K signaling pathway, growth, and metastasis of HeLa cells (Figure 6K,L). Compared with DP or LFA alone, DMFA had the best inhibitory effect on the PI3K‐Akt signaling pathway, thus leading to the best inhibition ability on the growth and metastasis of HeLa cells.

### 2.6 In Vivo Evaluation

**Figure 7:**
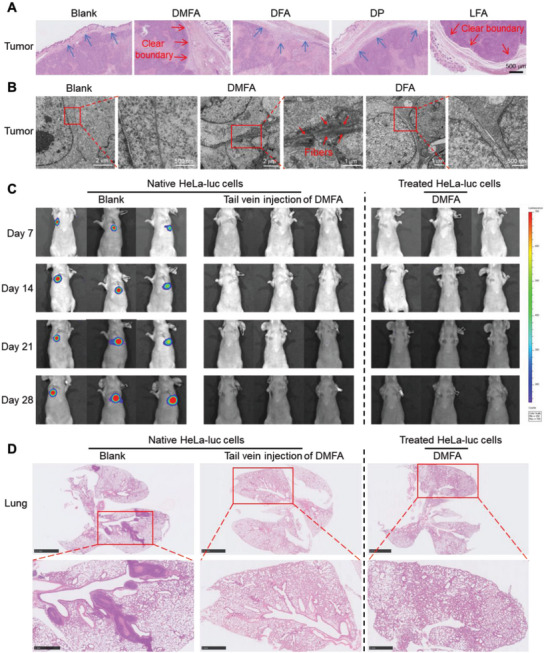
Inhibitory effect of DMFA on the growth and metastasis of HeLa cells in vivo. A) H&E‐stained tumor tissue sections from HeLa tumor‐bearing nude mice after various treatments. B) Bio‐TEM of tumor tissues from HeLa tumor‐bearing nude mice after various treatments. C) In vivo bioluminescence imaging of nude mice after receiving different treatments (*n* = 3 per group). D) H&E‐stained lungs of nude mice after receiving different treatments. The scale bars of the first line of images were 2.5 mm, and the scale bars of the enlarged images were 1 mm.

Encouraged by the satisfying effects in vitro, several animal models were developed to evaluate the therapeutic effect of DMFA on tumor growth and metastasis in vivo. First, to study the inhibitory effect of DFMA on tumor growth in vivo, we established HeLa tumor‐bearing xenografts of nude mice and divided them into five groups randomly. After the tumors grew to 100 mm^3^, different probes were conducted. As shown in Figure S45, Supporting Information, DP, LFA, and DFA could inhibit tumor growth to a certain extent (*p < 0.05) compared with the blank group, while there was a significant difference between DMFA and blank group (**p < 0.01), which indicated that ascribing to the combined treatments on the cell membrane of two ways, DMFA could inhibit the tumor growth to the most extent. Then, after treatment for 17 days, mice were sacrificed, then organs and tumors were collected for further examination. H&E staining showed that there was no obvious damage in the organs of the mice treated by DMFA (Figure S46, Supporting Information), which indicated that DMFA had less organ toxicity and side effects. As to H&E staining of tumors, it is found that the tumors treated with DMFA and LFA showed a clear tumor boundary, while the tumors in other groups showed a trend of outward expansion (**Figure** **7**A), ascribing to the formation of nanofibers to wrap the tumors. To further verify the formation of nanofibers from DMFA, Bio‐TEM studies on excised tumor sections were carried out. As shown in Figure 7B, Bio‐TEM images of the DMFA group showed abundant nanofibers on the tumor cells membrane compared with the blank group and DFA group, which meant that DMFA could be cut by MMP‐2 and transformed into nanofibers in situ. These animal experimental results suggested that DMFA could be cleaved by MMP‐2 in vivo and inhibited tumor growth.

Then, we established nude mice tumor metastasis model to detect the inhibitory effect of DMFA on tumor cell metastasis in vivo.^[^ ^19^ ^]^ Luciferase‐labeled HeLa (HeLa‐luc) cells were injected into nude mice via the tail vein. After 24 h, DMFA was injected through the tail vein every other day for 3 weeks. All mice were imaged every week with an in vivo imaging system for 4 weeks. As shown in Figure 7C (left side of dashed line), the fluorescence emitted by HeLa‐luc cells was detected in the blank group on the 7th day, and the fluorescence intensity gradually increased with time, indicating that metastatic foci of HeLa‐luc cells formed in the lungs of mice and gradually proliferated in the blank group. However, the fluorescence emitted by HeLa‐luc cells was not detected in DMFA‐treated mice, which illustrated that DMFA could effectively inhibit the metastasis of HeLa cells in vivo. H&E staining of the lung further proved that there was no metastasis in the lungs of the mice in the DMFA group, while there was obvious metastasis in the lungs of the mice in the blank group (Figure 7D, left side of the dashed line).

Finally, HeLa‐luc cells were incubated with DMFA, DP, and LFA in vitro and then injected into nude mice via the tail vein. All mice were imaged every week with an in vivo imaging system for a total of 4 weeks. During this period, no other treatments were performed on the mice. As shown in Figure 7C (right side of dashed line) and Figure S47, Supporting Information, all mice in DMFA and DP groups did not show metastatic foci in the lung, while one mouse in the LFA group showed metastatic foci in the lung at the third week. These data indicated that the ability of HeLa‐luc cells treated with DP to undergo metastasis was weaker than that of cells treated with LFA, and DMFA‐treated HeLa‐luc cells had no metastatic ability in vivo. Then H&E staining of the lung also proved that there was no metastasis in the lungs of the mice in the DMFA and DP group, while there was metastasis in the lungs of one mouse in the LFA group (Figure 7E, right side of the dashed line and Figure S48, Supporting Information). Through the verification of these animal experiments, DMFA could be digested by MMP‐2 and formed nanofibers in vivo. In addition, both DP and LFA could inhibit HeLa tumor growth and metastasis to a certain extent, but their combined treatment (DMFA) could more effectively inhibit HeLa tumor growth and metastasis in vivo.

## 3 Conclusion

To achieve the effective inhibition of tumor growth and metastasis, in this study, we proposed a smart transformable peptide‐conjugated probe DMFA with MMP‐2 cleavage‐induced morphological change. DMFA could assemble into nanoparticles initially with the exposure of the *α*‐helix peptide on the outside, thus, anchoring on the cell membrane rapidly and stably. Then DMFA in situ converted to DP with *α*‐helix structure and LFA with *β*‐sheet structure in response to MMP‐2. Combing the influx of Ca^2+^ and the disruption of the Na^+^/K^+^‐ATPase by two parts, the intracellular PI3K‐Akt signaling pathway could be inhibited. By ensuring the sufficient interaction sites between *α*‐helix and phospholipid bilayer and the precise formation of LFA‐based nanofibers outside the cell membranes, tumor growth and metastasis could be efficiently prevented. This approach of cleavage‐induced morphological change for peptide‐conjugated probes could provide not only a new strategy for efficient tumor therapy but also have the potential to deeply investigate the dynamics between the nanofiber formation and cellular internalization.

## 4 Experimental Section

### Materials and Instruments

Sodium ascorbate and copper bromide were purchased from Aladdin Biochemical Technology Co., Ltd (Shanghai, China). The peptides DMF, DF, LF, and DP (Table S1, Supporting Information) were customized by GL Biochem Ltd. (Shanghai, China). Recombinant human MMP‐2 was purchased from Sino Biology Inc (Beijing, China). MTT was purchased from Sangon Biotech Co., Ltd (Shanghai, China). Deionized water (18.2 MΩ cm) used in all experiments was purified with a Heal Force water purification system (Shanghai, China). MMP‐2 inhibitor APR100 was purchased from GLPBIO. Fura‐2 AM was purchased from Yeasen Biotechnology Co., Ltd (Shanghai, China). Human MMP‐2 ELISA Kit was purchased from Sangon Biotech Co., Ltd. LDH Assay Kit was purchased from Beyotime Biotechnology. Micro Na^+^/K^+^‐ATPase Assay Kit was purchased from Beijing Solarbio Science & Technology Co., Ltd. All other reagents were obtained from commercial sources and used without further purification.

HPLC of probes synthesis part was performed by Shimadzu LC‐20A HPLC under the test wavelength of 220 or 254 nm. The sample was dissolved in water solution or acetonitrile, applied on an Ultimate XB‐C18 column (10 µm, 250 × 10 mm), and eluted with a 50‐min gradient from 20% to 100% solvent B at 2 mL min^−1^, where solvent A was water (0.1% TFA solution) and solvent B was acetonitrile (0.1% TFA solution). All products were purified by HPLC to reach a purity of 95%. HRMS were obtained on a Thermo Scientific Q Exactive mass spectrometer system operating in an ESI‐Obitrap mode. UV–vis absorption spectra were tested on a Shimadzu UV‐2600 spectrometer. All fluorescence measurements were tested on an Edinburgh FS5 Fluorescence Spectrophotometer. CD spectra were taken on a Jasco J‐810. FTIR spectroscopy was obtained on a Nicolet iS5. CLSM images were obtained on a Zeiss LSM 880 confocal laser scanning microscope. MTT assay was obtained on an Infinite M200 PRO Microplate Reader (Tecan, Austria). The hydrodynamic size and zeta potential of all probes were detected by DLS using a Malvern Instruments Zetasizer Nano ZS90. TEM images were obtained with an FEI Talos F200X TEM instrument with an accelerating voltage of 200 kV. The sample was stained with 0.2% (w/v) phosphotungstic acid solution before the TEM test. SEM images were taken on Hitachi SU8010. Transwell images were obtained with Carl Zeiss Axio Scope A1. Bio‐TEM images were obtained with an FEI Talos F200X TEM instrument with an accelerating voltage of 200 kV.

### General Procedure for Enzymatic Assay

The HPLC column used in this part was a Welchrom C18 column (5 µm, 250 × 4.6 mm), and the sample was eluted with a 30‐min gradient from 20% to 100% solvent B at 1 mL min^−1^, where solvent A was water (0.1% TFA solution) and solvent B was acetonitrile (0.1% TFA solution).

Recombinant human MMP‐2 protein (0.25 mg mL^−1^ stock solution) was first activated in the buffer (0.05% Brij, 0.05% Calcium chloride, 0.79% Tris HCl, 1.17% Sodium chloride, pH 7.4) with 2.5 mm 4‐aminophenylmercuric acetate at 37 °C for 2 h. Then the probe (DMFA or DFA) solution (1 mm stock solution) was mixed with the activated enzyme solution. The final concentrations of the probe and MMP‐2 were 100 µm and 10 µg mL^−1^, respectively. The deactivation of MMP‐2 was conducted by incubating the protein at 65 °C for 20 min. Moreover, the reaction product was analyzed by HPLC and HRMS.

### Dynamic Light Scattering Assay

The concentration of DMFA, DFA, DP, and LFA was 10 µm and the hydrodynamic size of each probe was obtained by DLS.

For the test that the probe was incubated with MMP‐2, 10 µm DMFA or DFA was first mixed with the activated MMP‐2 solution, respectively. Then the hydrodynamic size of the reaction product was obtained by DLS.

### Transmission Electron Microscope

The morphology of various samples was observed by TEM (FEI Talos F200X). DMFA, DFA, DP, and LFA were dropped on a copper mesh, respectively, then removed the unnecessary liquid by a piece of filter paper after 1 min. Next, the phosphotungstic acid solution was dropped on this copper mesh to stain the samples for 1 min. After removing most of the phosphotungstic acid solution using the same method described above, the copper mesh was washed with deionized water three times.

For the test that the probe was incubated with MMP‐2, DMFA or DFA was first mixed with the activated MMP‐2 solution, respectively. Then, the reaction products were dropped on a copper mesh as the same method described above.

### Atomic Force Microscope

AFM experiments were performed on a Bruker MultiMode 8 instrument. DMFA, DFA, DP, and LFA were dropped on the cleaned mica surface for 10 min and the retained solution on the mica surface was removed by filter paper. The samples were dried under atmospheric conditions before measurement.

For the test that the probe was incubated with MMP‐2, DMFA or DFA was first mixed with the activated MMP‐2 solution, respectively. Then, the reaction products were dropped on the mica surface as the same method described above.

### Circular Dichroism Spectra

DMFA, DFA, DP, and LFA were dissolved in phosphate buffer solution (PBS)/trifluoroethanol (v:v = 1:1), respectively. The final concentration of probes was 20 µm. The second structure of each probe was obtained by Jasco J‐810 at room temperature with a cell length of 1 mm. All spectra were the average of three measurements.

For the test that the probe was incubated with MMP‐2, DMFA or DFA was mixed with the activated MMP‐2 solution, respectively. Then the reaction product was dissolved in PBS/trifluoroethanol (v:v = 1:1) and the second structure was obtained by Jasco J‐810 at room temperature with a cell length of 1 mm. All spectra were the average of three measurements.

### Fourier Transform Infrared Spectroscopy

Samples were dispersed in water to 10 µm and stood for a period of time. The liquid samples were tested by Nicolet iS5. All the results were averaged among 16 measurements and the background was deducted.

### Cell Culture

HeLa cells and MCF‐7 cells were cultured in Dulbecco's Modified Eagle Medium (DMEM). HeLa‐luc cells were cultured in Minimum Essential Medium (MEM). Each cell was finally cultured with 10% FBS and 1% penicillin‐streptomycin in a culture flask at 37 °C in a humidified atmosphere containing 5% CO_2_.

### HRMS Analysis of Cell Lysates

After incubating 1 × 10^7^ HeLa cells with 20 µm DMFA or DFA in the DMEM culture medium at 37 °C, cells were washed three times with PBS. The cells were collected and conducted repeated freezing and thawing at least ten times, and then sonicated on ice with a cell breaker at 20% power to obtain cell lysates. Then the cell lysate was analyzed by HRMS.

### Incubation of HeLa Cells with MMP‐2 Inhibitor ARP100

HeLa cells were incubated with 10 µm ARP100 for 24 h to decrease the expression of MMP‐2.

### CLSM Imaging

For CLSM imaging, cells were seeded into cell culture dishes at a density of 2 × 10^4^ in a growth medium. After overnight incubation, the cells were washed with phosphate‐buffered saline three times. A solution of the indicated probe in a medium was then added, and the cells were incubated in a 5% CO_2_ atmosphere at 37 °C for further usage. The supernatant was then discarded, and the cells were washed gently twice with PBS and immersed in a growth medium prior to optical imaging.

The fluorescence signals were detected by using a Zeiss LSM 880 confocal microscope with a 63× oil‐immersion objective. A 488 nm laser was chosen for the excitation of PyTPA, the emission was collected at 600–740 nm. A 543 nm laser was chosen for the excitation of PI, the emission was collected at 600–660 nm. A 633 nm laser was chosen for the excitation of DID, the emission was collected at 645–680 nm. A 405 nm laser was chosen for the excitation of Hoechst 33 258, the emission was collected at 410–460 nm.

### Multicellular Tumor Spheroids Experiment

At the bottom of 96‐well plates, 50 µL agarose (0.1% wt) was added. Then, the 96‐well plate was put into the 37 °C oven for 1 h. After sterilization, 2000 cells were seeded into each well and incubated for 4 days until the tumor spheroids were observed.

### SEM Images of Cell Surfaces

HeLa cells were cultured in glass‐bottom dishes for 12 h. DMFA, DFA, DP, and LFA (20 µm) were incubated with cells in DMEM at 37 °C, respectively. The cells were solidified with glutaraldehyde (2.5%) for 2 h and then coated with gold for 30 s.

### Cytotoxicity Assay

MTT assays were used to assess the cell viability of HeLa cells after incubation with the probes. The cells in 96‐well plates were incubated with the probes for a designated time. After incubation, the cells were washed with PBS. Then MTT in PBS solution was added to each well. After incubation for 4 h, the supernatant was discarded and the precipitate was dissolved in DMSO (100 µL) with gentle shaking. The absorbance of MTT at 570 nm was monitored by the Infinite M200 PRO microplate reader.

### Scratch Test

HeLa cells were plated into a culture dish and incubated until almost overgrown. Then, a 200 µL pipette tip was used to draw a straight line to create a wound. The cells were then incubated for another 24 or 48 h with DMEM containing different probes at 37 °C in a 5% CO_2_ humidified incubator. At 0, 24, or 48 h, the wound healing area was photographed under CLSM (Zeiss LSM 880 confocal microscope). The images were analyzed by image analysis software (ImageJ).

### Transwell Migration and Invasion Assay

In the migration experiment, 5 × 10^5^ cells were plated to the top chambers with serum‐free DMEM containing different probes of transwell (Corning) without Matrigel. The bottom chamber was added with 600 µL of medium containing 20% FBS. After 24 h, the top chambers were washed with PBS, fixed with paraformaldehyde for 20 min, and stained with 0.1% crystal violet. The cells that did not migrate were wiped away and the cells that passed through the membrane were counted through microscopy (Carl Zeiss Axio Scope A1).

In the invasion experiment, 6 × 10^5^ cells were plated to the top chambers with serum‐free DMEM containing different probes of transwell coated with Matrigel (BD Biosciences). The bottom chamber was added with 600 µL of medium containing 20% FBS. After 24 h, the top chambers were washed with PBS, fixed with paraformaldehyde for 20 min, and stained with 0.1% crystal violet. The cells that did not invade were wiped away and the cells that passed through the membrane were counted through microscopy (Carl Zeiss Axio Scope A1).

### Transcriptome Test

HeLa cells were plated in culture dishes and incubated for 24 h. Then the dishes were washed with PBS and added with DMEM or DMEM containing 20 µm DMFA. After another 24 h at 37 °C in a 5% CO_2_ humidified incubator, the total RNA was extracted using TRIzol reagent. In total, three transcriptomes of native HeLa cells and HeLa cells incubated with DMFA were extracted. All samples were delivered to Shanghai Personalbio Technology Co., Ltd to perform RNA sequencing.

### In Vivo Antitumor Study

Female BALB/c nude mice (4 weeks old, ≈20 g body weight) were purchased from Beijing Vital River Laboratory Animal Technology Co., Ltd., China. Animal care and handling procedures were in agreement with the guidelines evaluated and approved by the Ethics Committee of Tongji Hospital, Tongji Medical College, Huazhong University of Science and Technology (Ethics No. TJH‐201903002). For the therapy of cancer, HeLa cells (2.5 × 10^6^) were injected into the underarm of mice.

The HeLa‐tumor‐bearing nude mice were divided into five groups (*n* = 3 per group) randomly. All the mice were treated by hypodermic injection around the tumor every other day with 100 µL of probes (400 µm) in PBS. The tumor sizes were measured by a caliper every other day, and the tumor volume was calculated as (tumor length) × (tumor width)^2^/2. Relative tumor volumes were calculated as *V*/*V*_0_ (*V*_0_ was the tumor volume when the treatment was initiated). On the 17th day, all the mice were sacrificed.

To evaluate the systemic toxicity, HeLa tumor‐bearing nude mice were sacrificed on the 17th day. Afterward, the tumor and major organs, including the heart, liver, lung, spleen, and kidney, were collected and observed with H&E staining. In addition, afterward, tumors of group PBS, DMFA, and DFA were collected and observed with Bio‐TEM.

### Tumor Metastasis Model

For DMFA inhibiting the formation of lung metastasis in vivo, nude mice were divided into two groups (*n* = 3 per group) randomly. 1 × 10^7^ native HeLa‐luc cells were injected into each mouse through the tail vein. After 24 h, three mice of the DMFA group were injected with 100 µL of 40 µm DMFA via the tail vein every other day for 3 weeks, and the other three mice of the blank group received an equal volume of PBS. All mice were imaged with an in vivo imaging system (IVIS spectrum, Perkin–Elmer) every week for 4 weeks. Images were obtained 15 min after an intraperitoneal injection of 150 mg Kg^−1^ D‐luciferin potassium salt and analyzed with image analysis software (living image).

To evaluate the ability of cells pretreated with probes to form lung metastasis in vivo, nude mice were divided into four groups (*n* = 3 per group) randomly. 1 × 10^7^ HeLa‐luc cells preincubated with different probes (40 µm) for 24 h were injected into mice via tail vein. All mice were imaged with an in vivo imaging system (IVIS spectrum, Perkin‐Elmer) every week for 4 weeks. Images were obtained 15 min after an intraperitoneal injection of 150 mg Kg^−1^ D‐luciferin potassium salt and analyzed with image analysis software (living image).

To evaluate the metastatic foci formed by HeLa‐luc cells in the lung, nude mice of the tumor metastasis model were sacrificed on the 30th day. Afterward, lungs were collected and observed with H&E staining.

### Statistical Analysis

All experiments were performed at least three times and results were given as mean ± standard deviation. Data analysis was conducted by using ImageJ, Microsoft Excel, or OriginPro software. The statistical analysis was conducted by using a two‐sided Student's *t*‐test. The differences were considered statistically significant as follows: *p < 0.05, **p < 0.01, ***p < 0.001, ****p < 0.0001.

## Conflict of Interest

The authors declare no conflict of interest.

## Supporting information

Supporting Information

## Data Availability

The data that support the findings of this study are available in the supplementary material of this article.
