# Supplementary material for: A Peptide‐Conjugated Probe with Cleavage‐Induced Morphological Change for Treatment on Tumor Cell Membrane
Source: Adv Sci (Weinh). 2023 Feb 15;10(11):2207228. doi: 10.1002/advs.202207228 (PMC10104630; doi:10.1002/advs.202207228)
Supplement: Supplementary file 1 — Supporting Information [file ADVS-10-2207228-s001.pdf]

ADVANCED SCIENCE
Open Access

Supporting Information

for *Adv. Sci.*, DOI 10.1002/advs.202207228

A Peptide-Conjugated Probe with Cleavage-Induced Morphological Change for Treatment on Tumor Cell Membrane

*Wei Zhang, Jing-Jing Hu, Rui Liu, Jun Dai, Lizhen Yuan, Yiheng Liu, Bochao Chen, Mingxing Gong, Fan Xia and Xiaoding Lou**

Supporting Information

A peptide-conjugated probe with cleavage-induced morphological change for treatment on tumor cell membrane

Wei Zhang[†], *Jing-Jing Hu*[†], *Rui Liu, Jun Dai, Lizhen Yuan, Yiheng Liu, Bochao Chen, Mingxing Gong, Fan Xia, and Xiaoding Lou**

W. Zhang, Prof. J. Hu, Prof. R. Liu, L. Yuan, Y. Liu, B. Chen, Dr. M. Gong, Prof. F. Xia, Prof. X. Lou
State Key Laboratory of Biogeology and Environmental Geology
Engineering Research Center of Nano-Geomaterials of Ministry of Education
Faculty of Materials Science and Chemistry
China University of Geosciences
Wuhan 430074, China
E-mail: louxiaoding@cug.edu.cn

Dr. J. Dai
Department of Obstetrics and Gynecology
Tongji Hospital
Tongji Medical College, Huazhong University of Science and Technology
Wuhan 430030, China

[†] W. Zhang and J. J. Hu contributed equally to this work.

Synthesis of PyTPA

PyTPA was synthesized according to a published method. HRMS (ESI) m/z: $[M]^+$ calcd, 601.3074; found, 601.3073 (Figure S1).

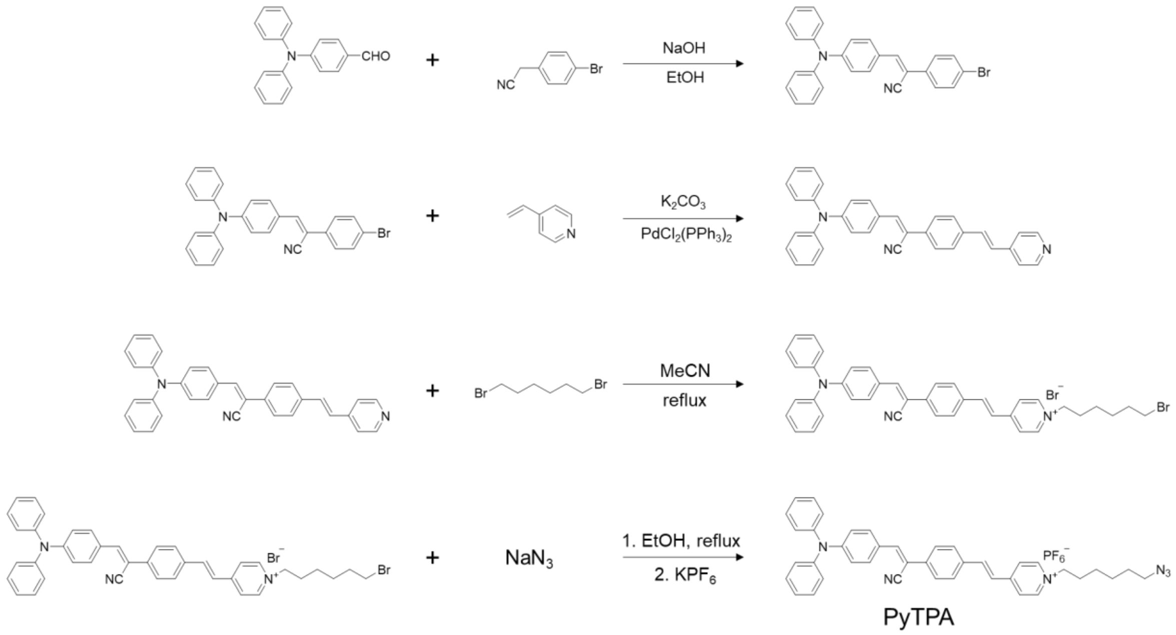

Scheme S1. The synthetic route of PyTPA.

Synthesis of DMFA

PyTPA (11.19 mg, 15 μmol, 1.5 equiv.), DMF (48.04 mg, 10 μmol, 1.0 equiv.), sodium ascorbate (3.96 mg, 20 μmol, 2.0 equiv.), and CuBr (3.59 mg, 25 μmol, 2.5 equiv.) were dissolved in DMSO/H_2O (v/v=1:1) and stirred at room temperature under the protection of nitrogen. The reaction was monitored by HPLC. After the reaction was completed, the crude product was separated by HPLC. The separated product was freeze-dried to afford a red solid and then tested by HRMS. HRMS (ESI) m/z: $[M+6H]^{7+}$ calcd, 773.1816; found, 773.1811 (Figure S2).

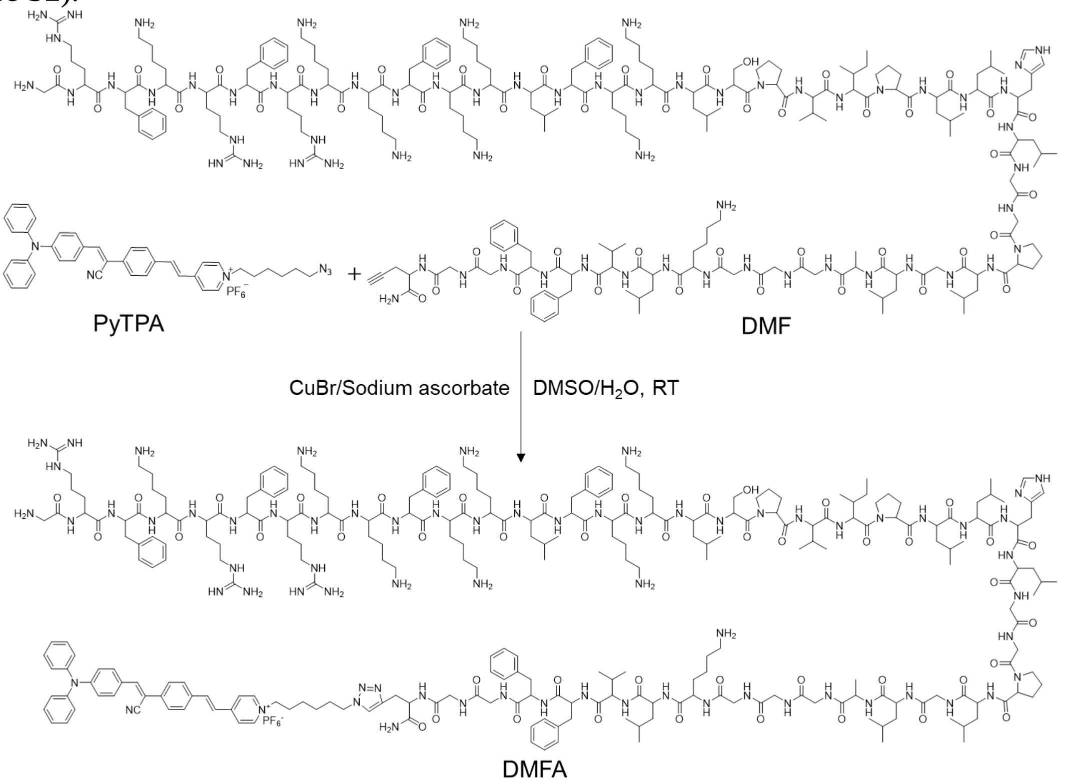

Scheme S2. The synthetic route of DMFA.

Synthesis of DFA

PyTPA (11.19 mg, 15 μmol, 1.5 equiv.), DF (41.83 mg, 10 μmol, 1.0 equiv.), sodium ascorbate (3.96 mg, 20 μmol, 2.0 equiv.), and CuBr (3.59 mg, 25 μmol, 2.5 equiv.) were dissolved in DMSO/H_2O (v/v=1:1) and stirred at room temperature under the protection of nitrogen. The reaction was monitored by HPLC. After the reaction was completed, the crude product was separated by HPLC. The separated product was freeze-dried to afford a red solid and then tested by HRMS. HRMS (ESI) m/z: $[M+5H]^{6+}$ calcd, 798.1533; found, 798.3240. $[M+6H]^{7+}$ calcd, 684.2754; found, 684.2780. $[M+7H]^{8+}$ calcd, 598.8669; found, 598.8686. $[M+8H]^{9+}$ calcd, 532.4381; found, 532.4403 (Figure S3).

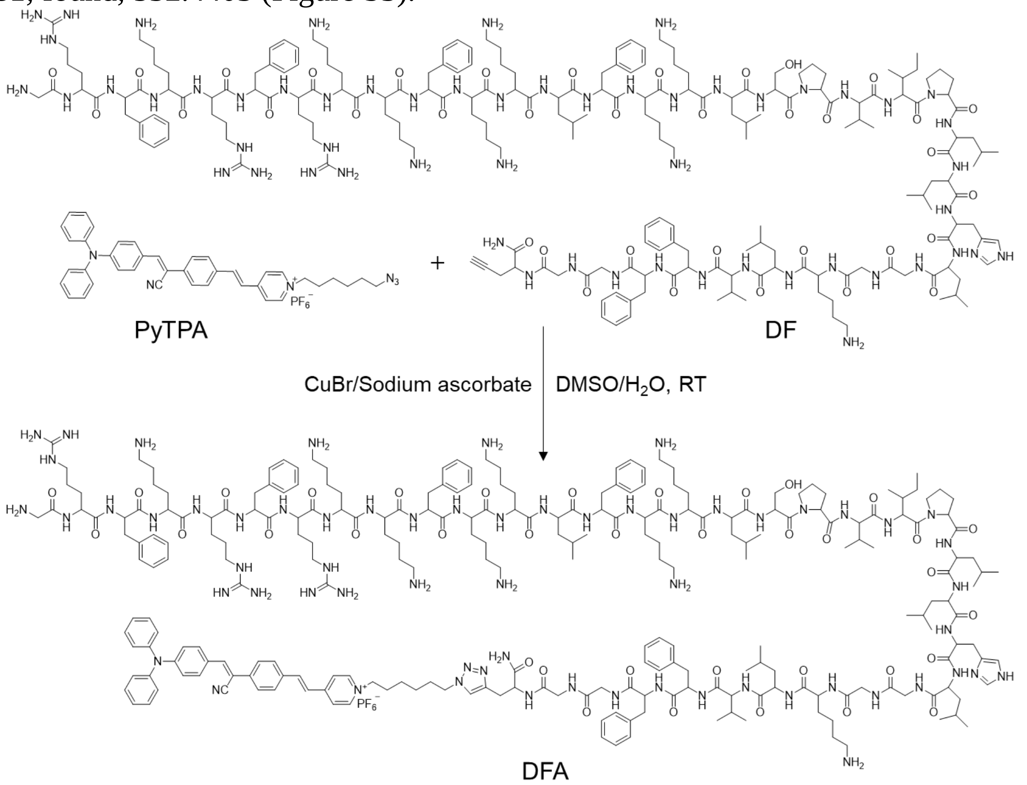

Scheme S3. The synthetic route of DFA.

Synthesis of LFA

PyTPA (11.19 mg, 15 μmol, 1.5 equiv.), LF (12.16 mg, 10 μmol, 1.0 equiv.), sodium ascorbate (3.96 mg, 20 μmol, 2.0 equiv.) and CuBr (3.59 mg, 25 μmol, 2.5 equiv.) were dissolved in DMSO/H_2O (v/v=1:1) and stirred at room temperature under the protection of nitrogen. The reaction was monitored by HPLC. After the reaction was completed, the crude product was separated by HPLC. The separated product was freeze-dried to afford a red solid and then tested by HRMS. HRMS (ESI) m/z: $[M+H]^{2+}$ calcd,909.4975; found, 909.4962. $[M+2H]^{3+}$ calcd, 606.6676; found, 606.6662 (Figure S4).

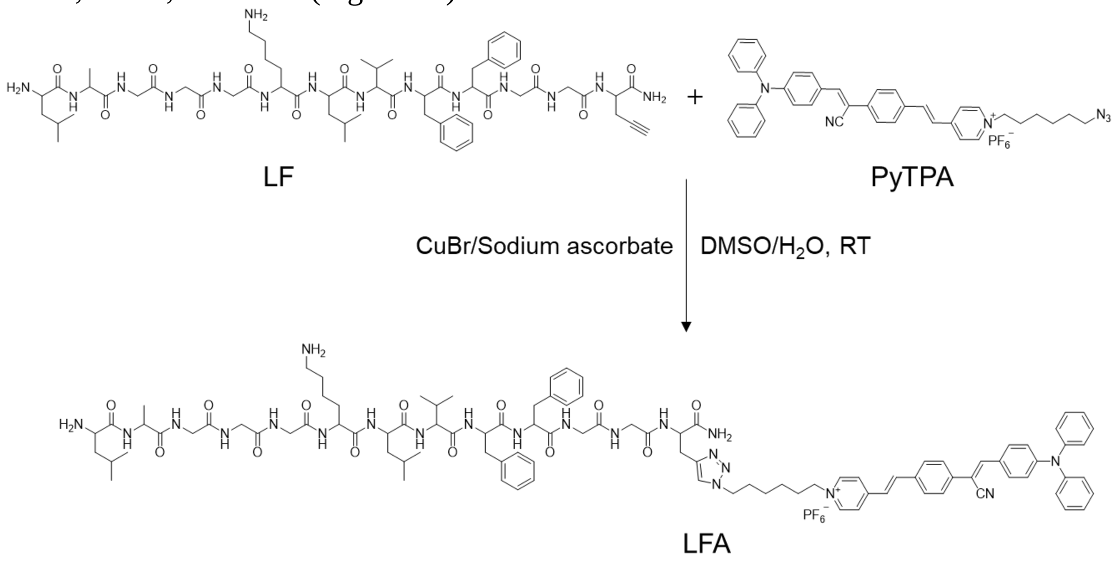

Scheme S4. The synthetic route of LFA.

Species	Peptide sequence (N ~ C)
**DMFA**	GRFKRFRKKFKKLFKKLSPVIPLLHLGGPLGLAGGGKLVFFGGPra-NH_2 (PyTPA)
**DFA**	GRFKRFRKKFKKLFKKLSPVIPLLHLGGKLVFFGGPra-NH_2 (PyTPA)
**DP**	GRFKRFRKKFKKLFKKLSPVIPLLHLGGPLG
**LFA**	LAGGGKLVFFGGPra-NH_2 (PyTPA)
**DMF**	GRFKRFRKKFKKLFKKLSPVIPLLHLGGPLGLAGGGKLVFFGGPra-NH_2
**DF**	GRFKRFRKKFKKLFKKLSPVIPLLHLGGKLVFFGGPra-NH_2
**LF**	LAGGGKLVFFGGPra-NH_2
**DP-DEAC**	DEAC-GRFKRFRKKFKKLFKKLSPVIPLLHLGGPLG

Table S1. Peptide sequences used in this study.

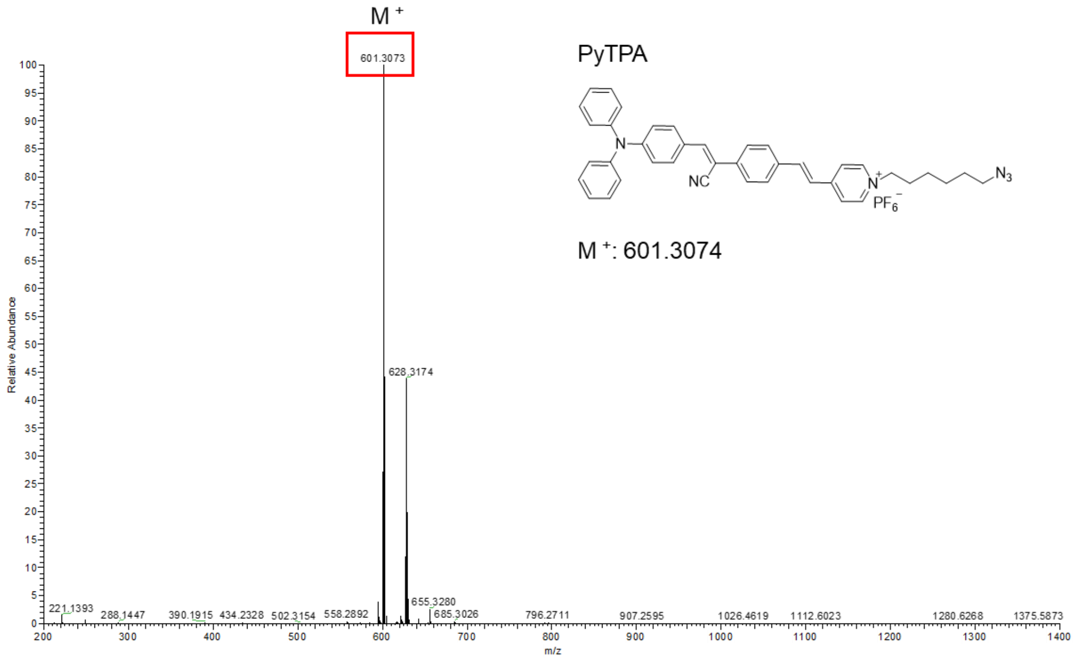

Figure S1. HRMS spectrum of compound PyTPA.

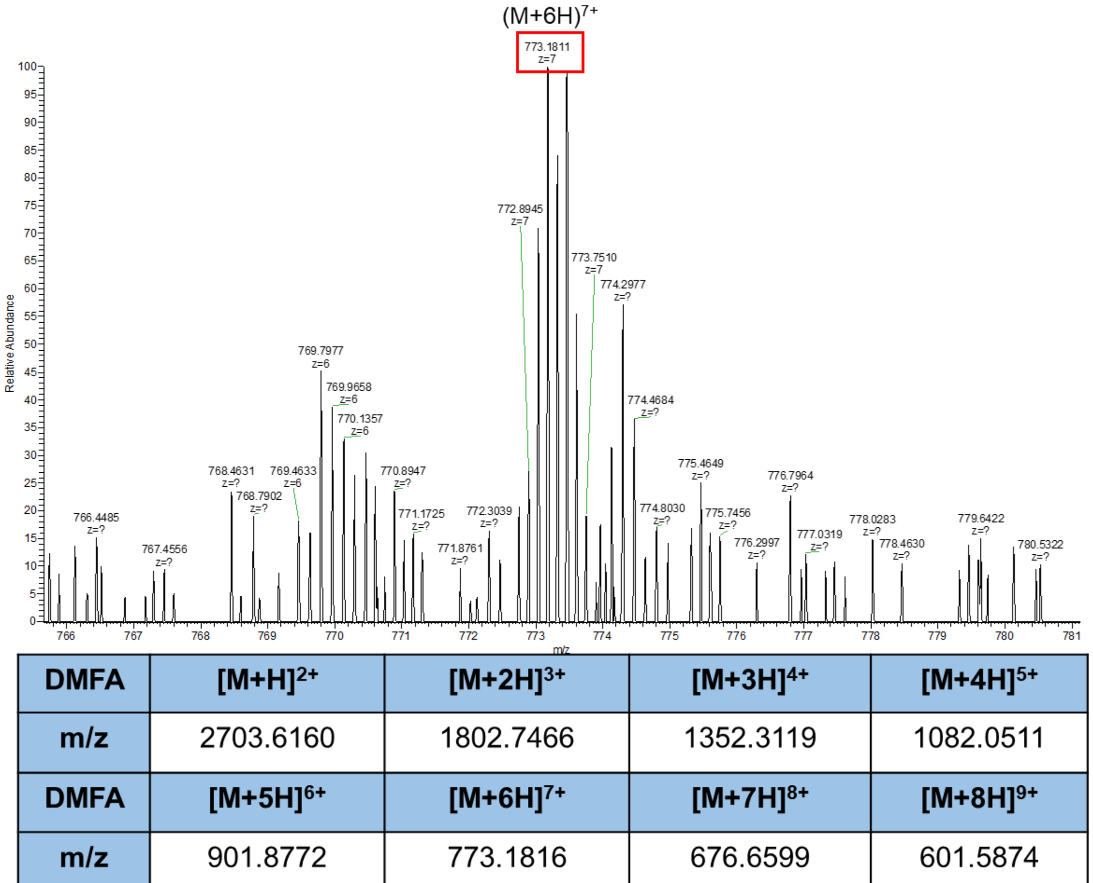

DMFA	[M+H]$^{2+}$	[M+2H]$^{3+}$	[M+3H]$^{4+}$	[M+4H]$^{5+}$
m/z	2703.6160	1802.7466	1352.3119	1082.0511
DMFA	[M+5H]$^{6+}$	[M+6H]$^{7+}$	[M+7H]$^{8+}$	[M+8H]$^{9+}$
m/z	901.8772	773.1816	676.6599	601.5874

Figure S2. HRMS spectrum of DMFA.

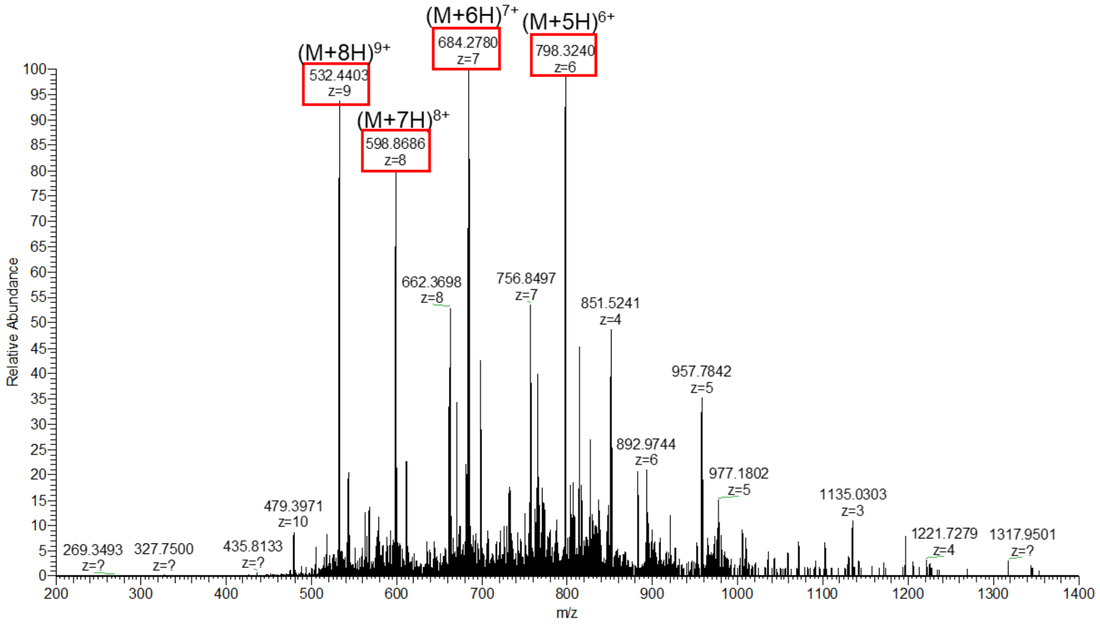

DFA	[M+H]$^{2+}$	[M+2H]$^{3+}$	[M+3H]$^{4+}$	[M+4H]$^{5+}$
m/z	2392.4441	1595.2987	1196.7260	957.5824
DFA	[M+5H]$^{6+}$	[M+6H]$^{7+}$	[M+7H]$^{8+}$	[M+8H]$^{9+}$
m/z	798.1533	684.2754	598.8669	532.4381

Figure S3. HRMS spectrum of DFA.

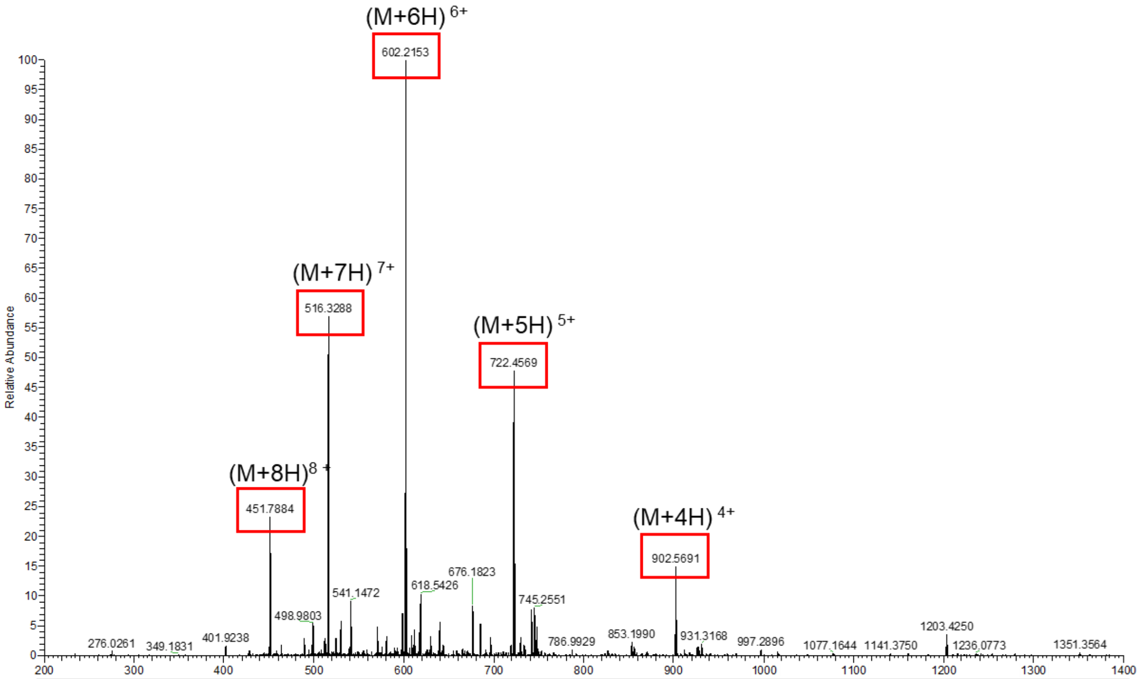

DP	[M+H]$^{+}$	[M+2H]$^{2+}$	[M+3H]$^{3+}$	[M+4H]$^{4+}$
m/z	3607.2555	1804.1317	1203.0904	902.5694
DP	[M+5H]$^{5+}$	[M+6H]$^{6+}$	[M+7H]$^{7+}$	[M+8H]$^{8+}$
m/z	722.2574	602.0491	516.1861	451.7888

Figure S4. HRMS spectrum of DP.

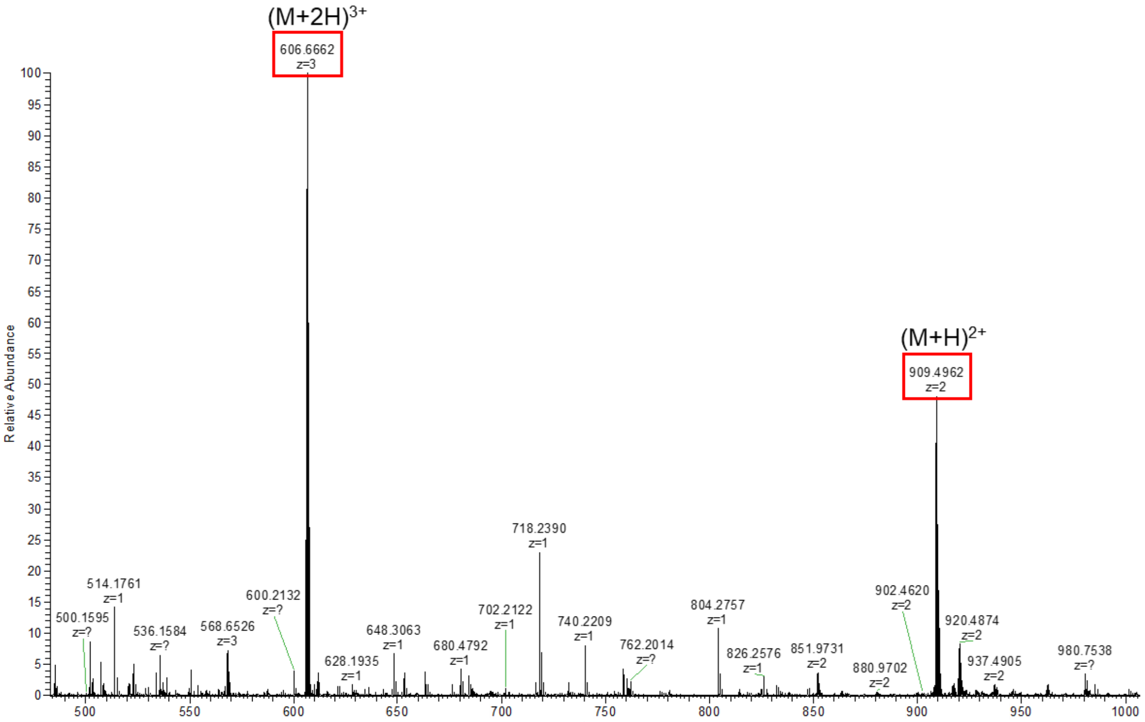

LFA	[M+H]$^{2+}$	[M+2H]$^{3+}$	[M+3H]$^{4+}$	[M+4H]$^{5+}$
m/z	909.4975	606.6676	455.2527	364.4037
LFA	[M+5H]$^{6+}$	[M+6H]$^{7+}$	[M+7H]$^{8+}$	[M+8H]$^{9+}$
m/z	303.8377	260.5763	228.1303	202.8944

Figure S5. HRMS spectrum of LFA.

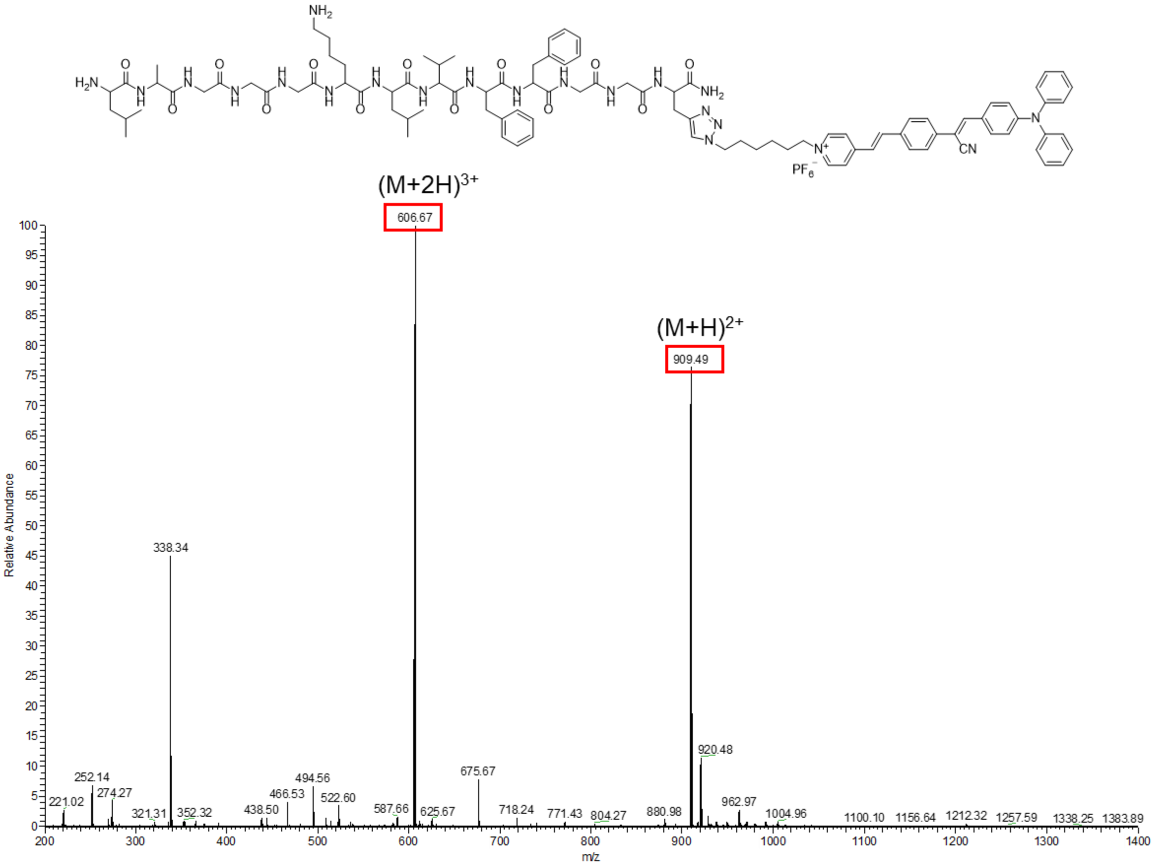

Figure S6. HRMS spectrum of enzymatic hydrolysis product of DMFA after incubation with MMP-2 for 2 h. The chemical structure of hydrolysis product was exhibited. The peptide sequence from N-C was LAGGGKLVFFGGPra-NH_2 (PyTPA).

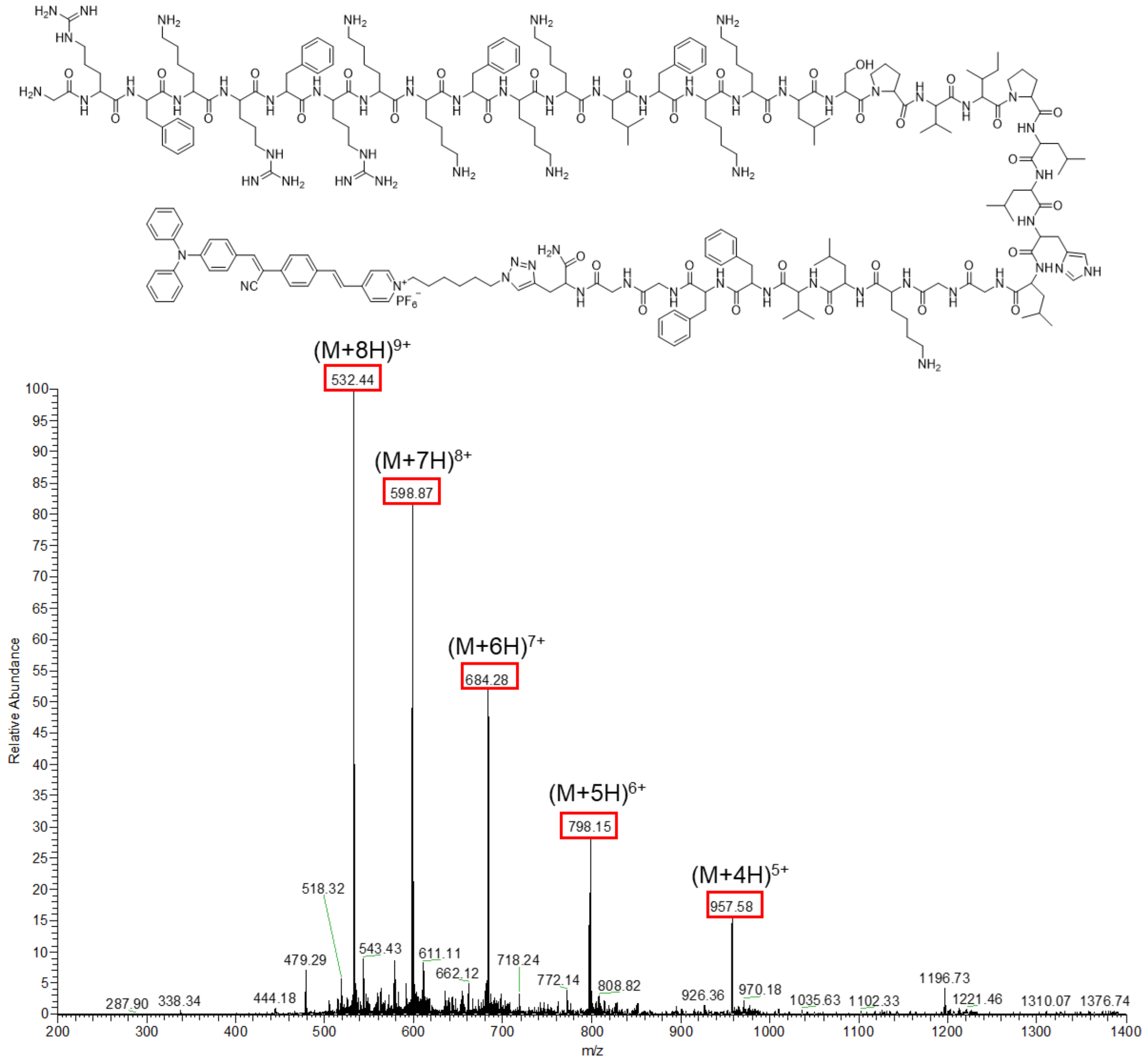

Figure S7. HRMS spectrum of enzymatic hydrolysis product of DFA after incubation with MMP-2 for 2 h. The chemical structure of hydrolysis product was exhibited. The peptide sequence from N-C was GRFKRFRKKFKKLFKKLSPVIPLLHLGGKLVFFGGPra-NH_2 (PyTPA).

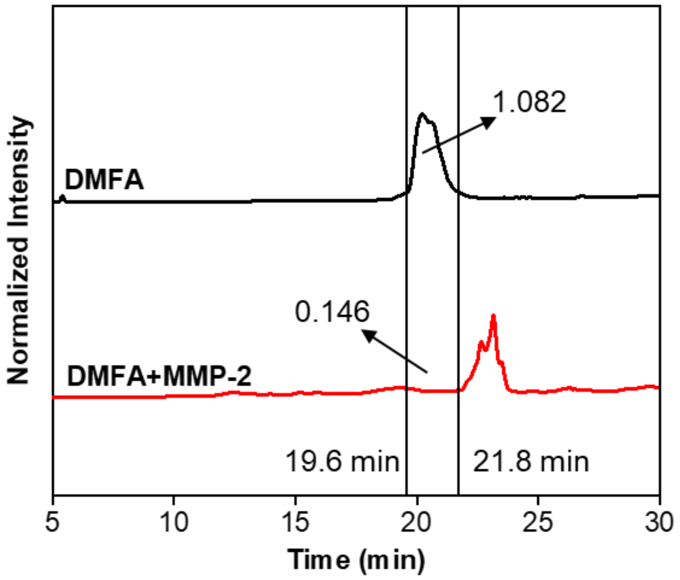

Figure S8. Calculation of digestion efficiency. For the DMFA curve, the peak area from 19.6 to 21.8 min was 1.082 through integration. For the DMFA+MMP-2 curve, the peak area from 19.6 to 21.8 min was 0.146 through integration. The digestion efficiency was (1.082-0.146)/1.082*100 %=86.5 %. Data was processed by OriginPro.

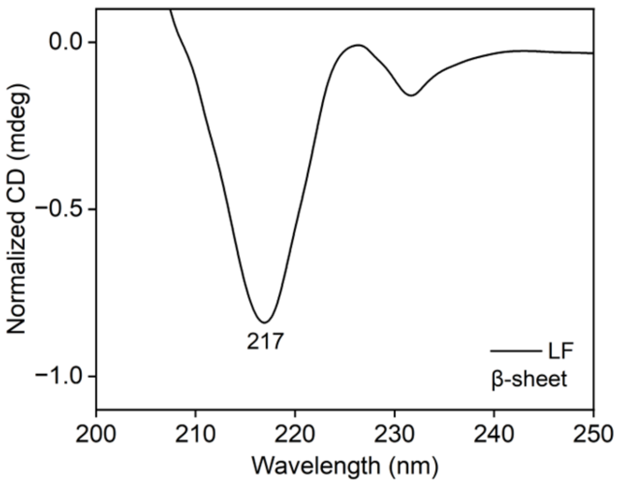

Figure S9. CD spectrum of LF. The concentration of the LF was 20 μM. The single negative signal at 217 nm represented the β-sheet structure of LF.

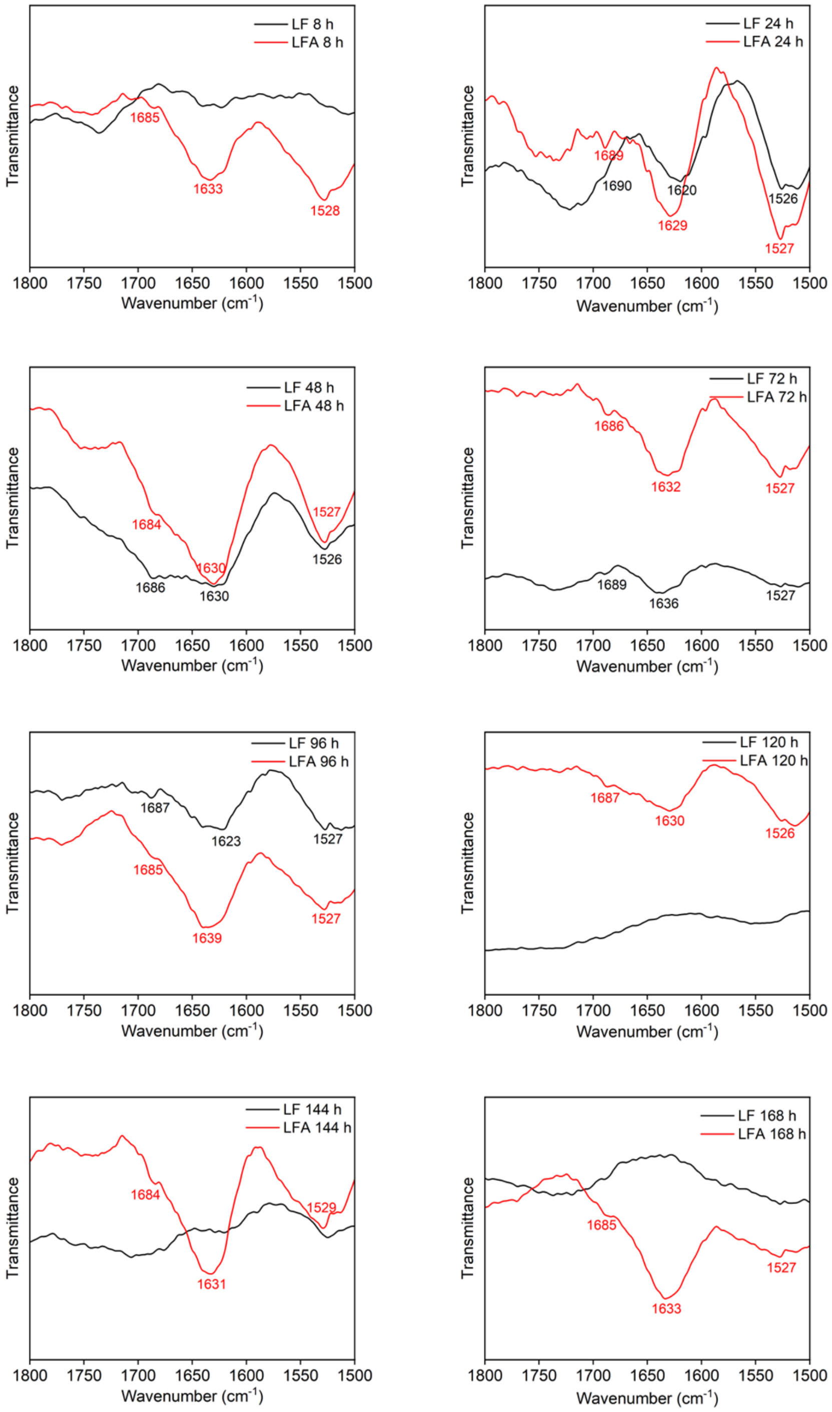

Figure S10. FTIR of LF and LFA with different times. The concentrations of LF and LFA were 10 μM. The antiparallel β-sheet conformation of LFA was detected from 8 h to 168 h, but that of LF was detected from 24 h to 96 h, indicating that the introduction of PyTPA was beneficial to the formation of antiparallel β-sheet structure.

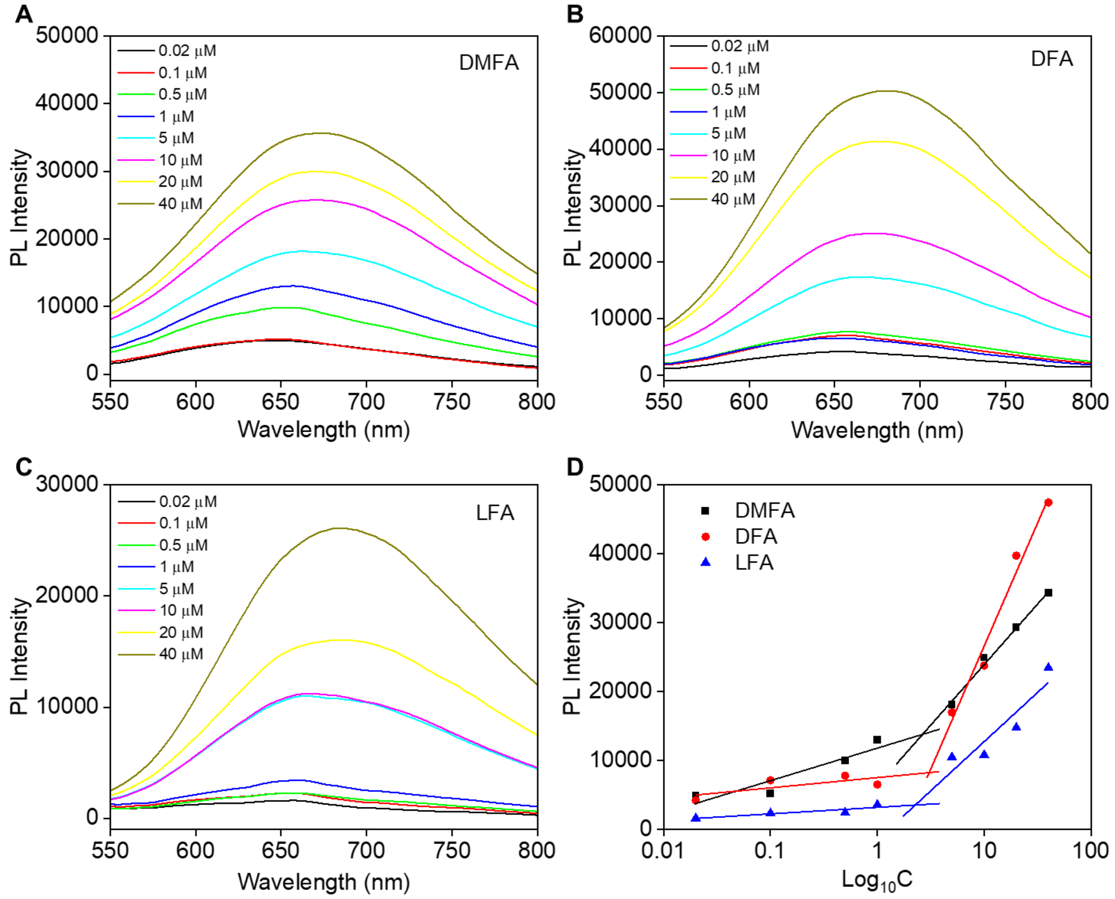

Figure S11. Fluorescence spectra of A) DMFA, B) DFA, and C) LFA with different concentrations. D) Critical micelle concentration (CMC) assay of DMFA, DFA, and LFA. The lowest concentration for DMFA, DFA, and LFA forming self-assembled structure was 2.69 µM, 3.09 µM, and 2.29 µM, respectively.

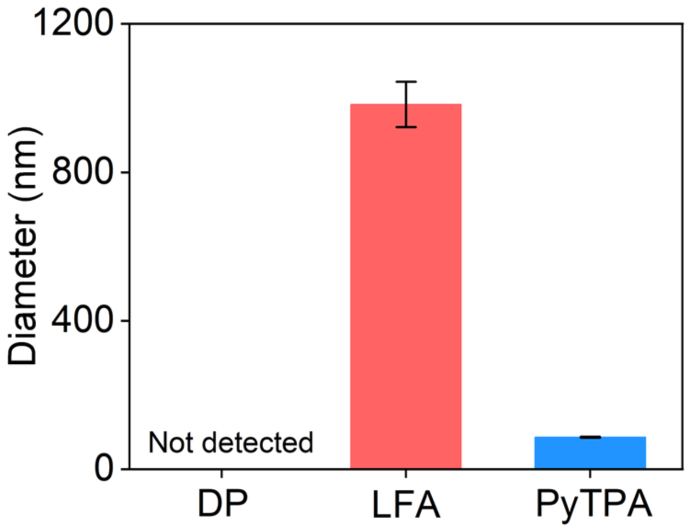

Figure S12. Hydrodynamic sizes of DP, LFA, and PyTPA (n=3). The concentrations of DP, LFA, and PyTPA were 10 μM. Data were presented as mean ± SD.

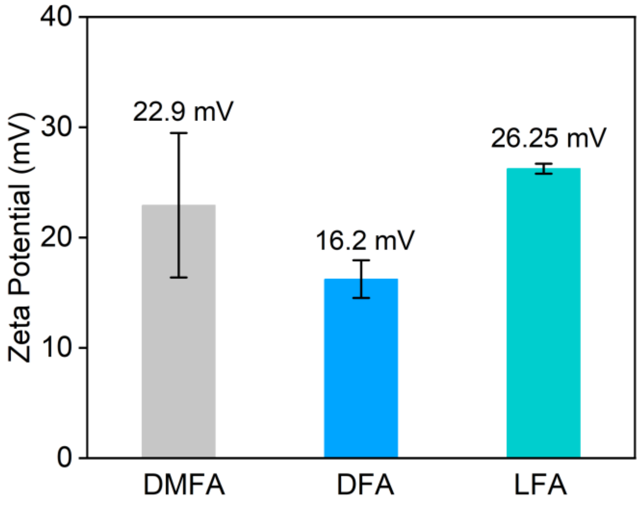

Figure S13. Zeta potentials assay. Zeta potentials of DMFA, DFA, and LFA in DMSO/water mixture (v/v=1:99) (n=3). The concentrations of DMFA, DFA, and LFA were 10 μM. Data was presented as mean ± SD.

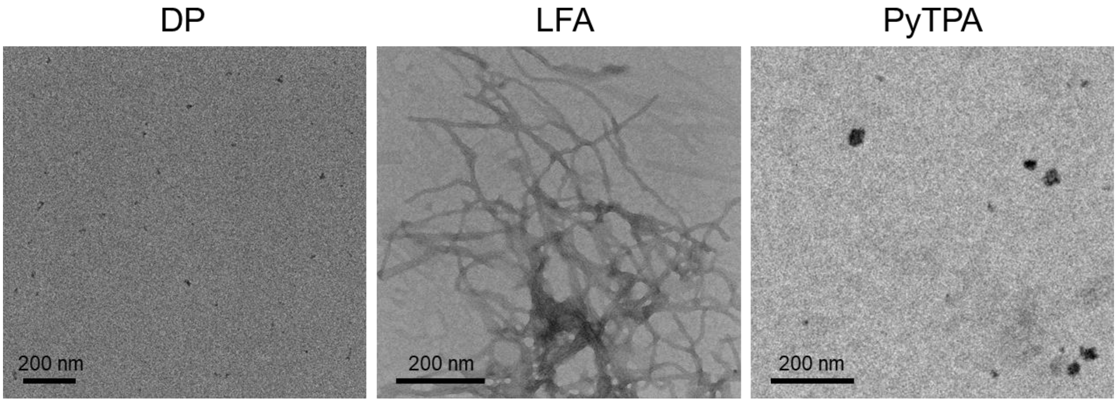

Figure S14. TEM images of DP, LFA, and PyTPA. The concentrations of DP, LFA, and PyTPA were 10 μM.

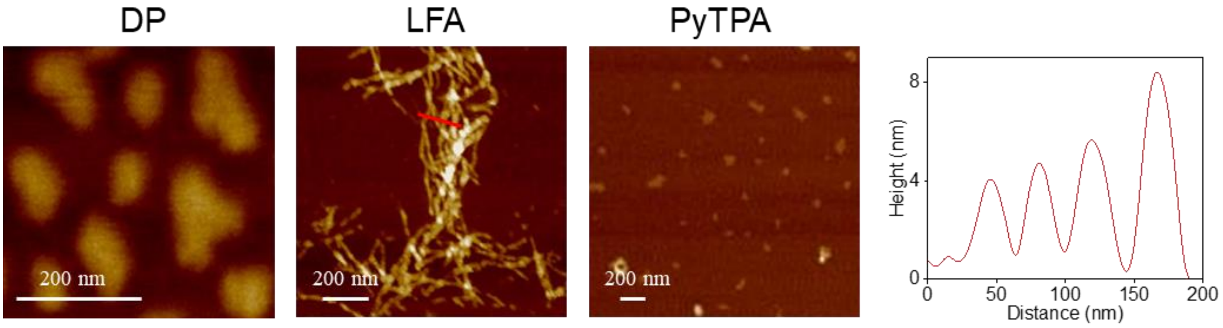

Figure S15. AFM images of DP, LFA, and PyTPA. The concentrations of DP, LFA, and PyTPA were 10 μM. Inset panels showed the height of the selected nanofibers. Through the DLS data, TEM images, and AFM images, PyTPA could form nanoparticles with a size about 80 nm.

Cell Line	Expression of MMP-2 enzyme mRNA
MCF-7 cell	0.057
HeLa cell	4.263

Figure S16. Expression of MMP-2 enzyme gene in different cancer cell lines. Data from Cancer Cell Line Encyclopedia (https://portals.broadinstitute.org/ccle/about).

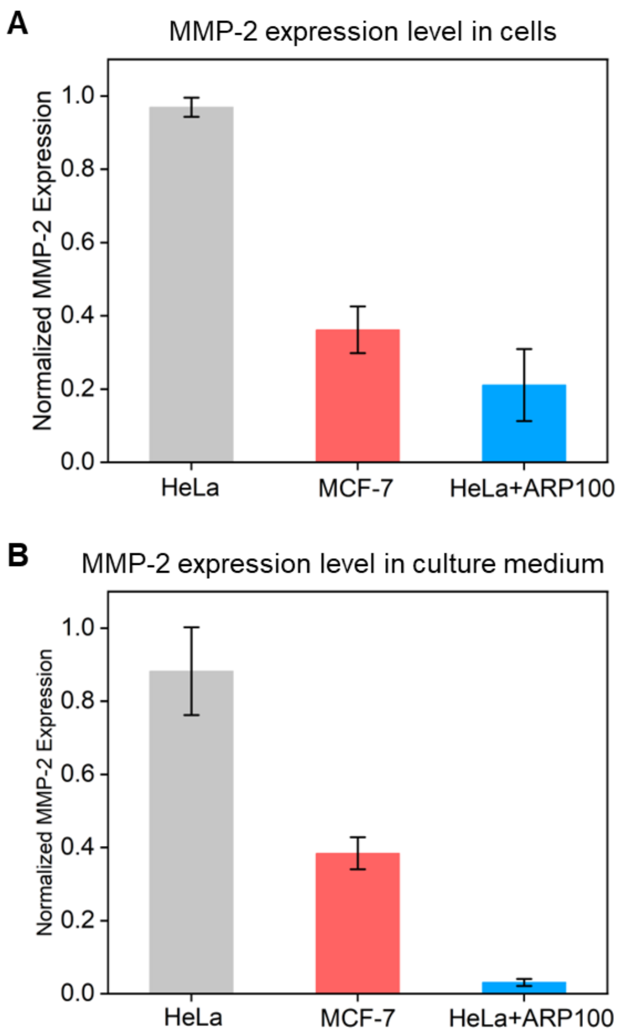

Figure S17. MMP-2 expression level assay. MMP-2 expression level in A) cells and B) cell culture medium (n=3). The cells included HeLa cells, MCF-7 cells, and HeLa cells after incubation with ARP100 for 24 h. The cell culture medium included the culture medium of HeLa cells, MCF-7 cells, and HeLa cells after incubation with ARP100 for 24 h. The concentration of ARP100 was 10 μM. Data were presented as mean ± SD. Data were tested by ELISA Kit purchased from Sangon Biotech Co., Ltd.

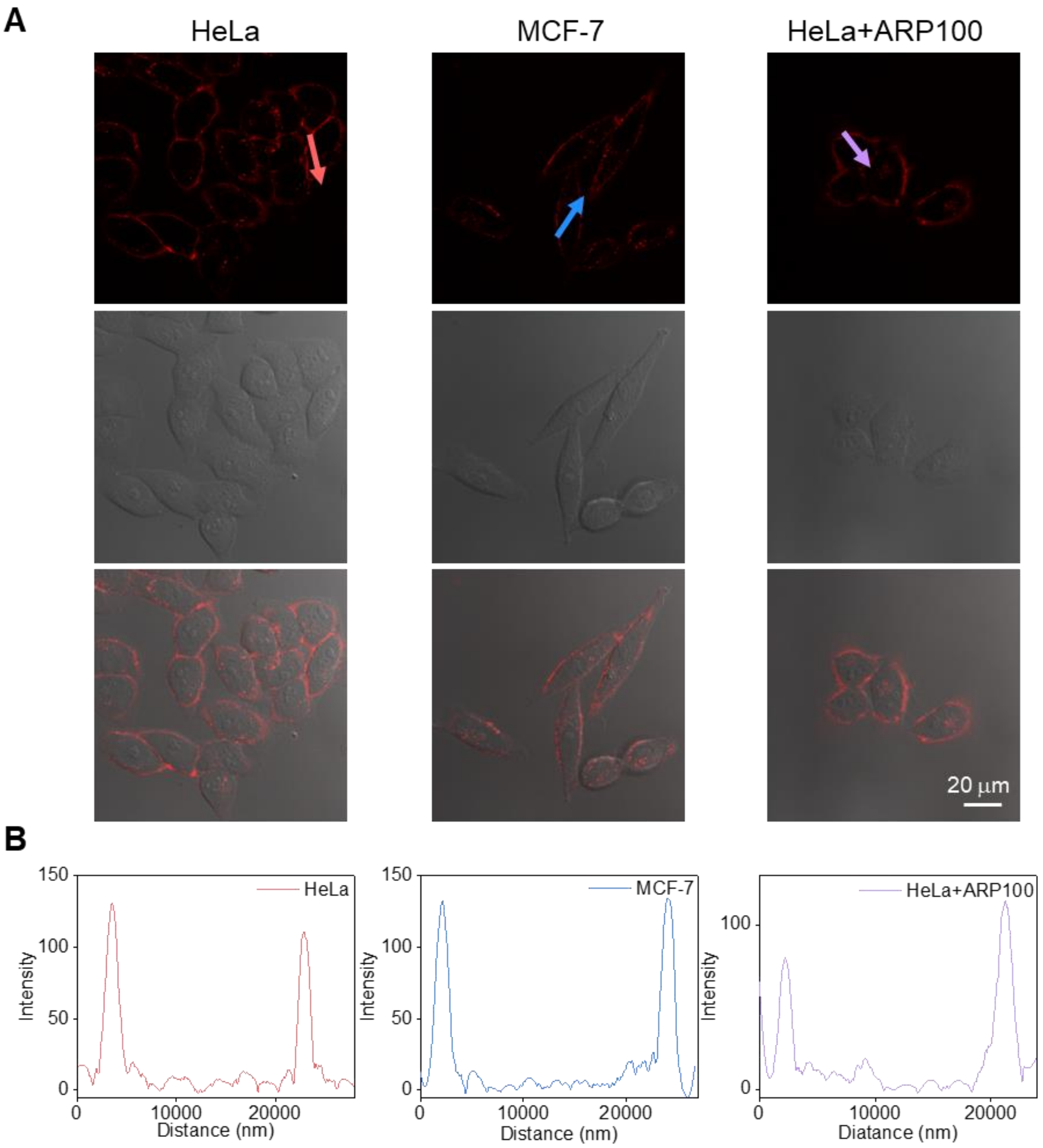

Figure S18. CLSM images of HeLa cells, MCF-7 cells, and HeLa+ARP100 incubated with LFA. A) CLSM images and B) intensity of HeLa cells, MCF-7 cells, and HeLa+ARP100 incubated with LFA (20 μM) for 4 h. The linear region across the HeLa cells, MCF-7 cells, and HeLa cells+ARP100 were marked by red arrows, blue arrows, and purple arrows respectively. A 488 nm laser was chosen for the excitation of LFA, the emission was collected at 600-740 nm (red fluorescence channel).

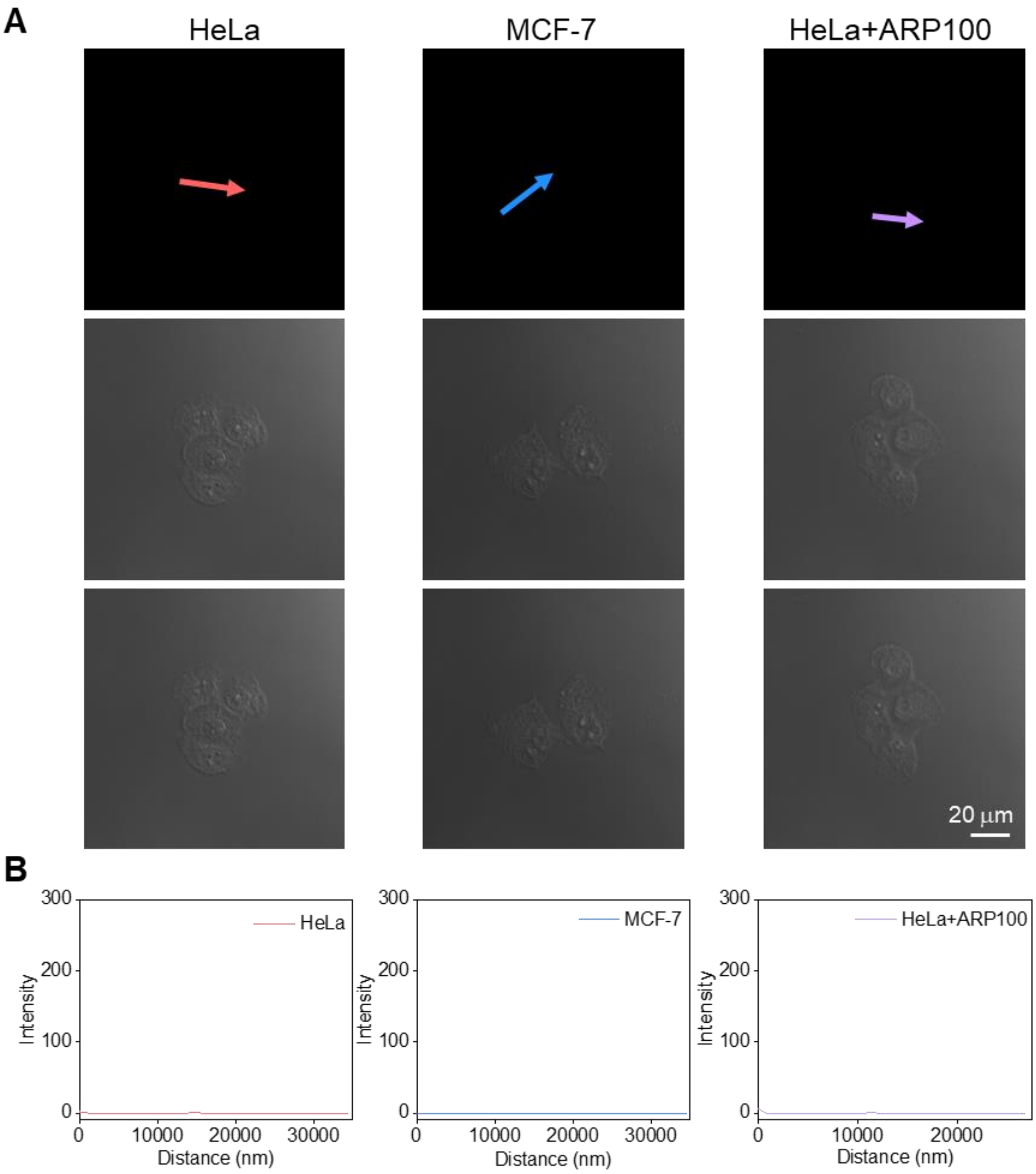

Figure S19. CLSM images of HeLa cells, MCF-7 cells, and HeLa+ARP100 incubated with DP. A) CLSM images and B) intensity of HeLa cells, MCF-7 cells, and HeLa+ARP100 incubated with DP (20 μM) for 4 h. The linear region across the HeLa cells, MCF-7 cells, and HeLa cells+ARP100 were marked by red arrows, blue arrows, and purple arrows respectively. A 488 nm laser was chosen for the excitation of DP, the emission was collected at 600-740 nm (red fluorescence channel).

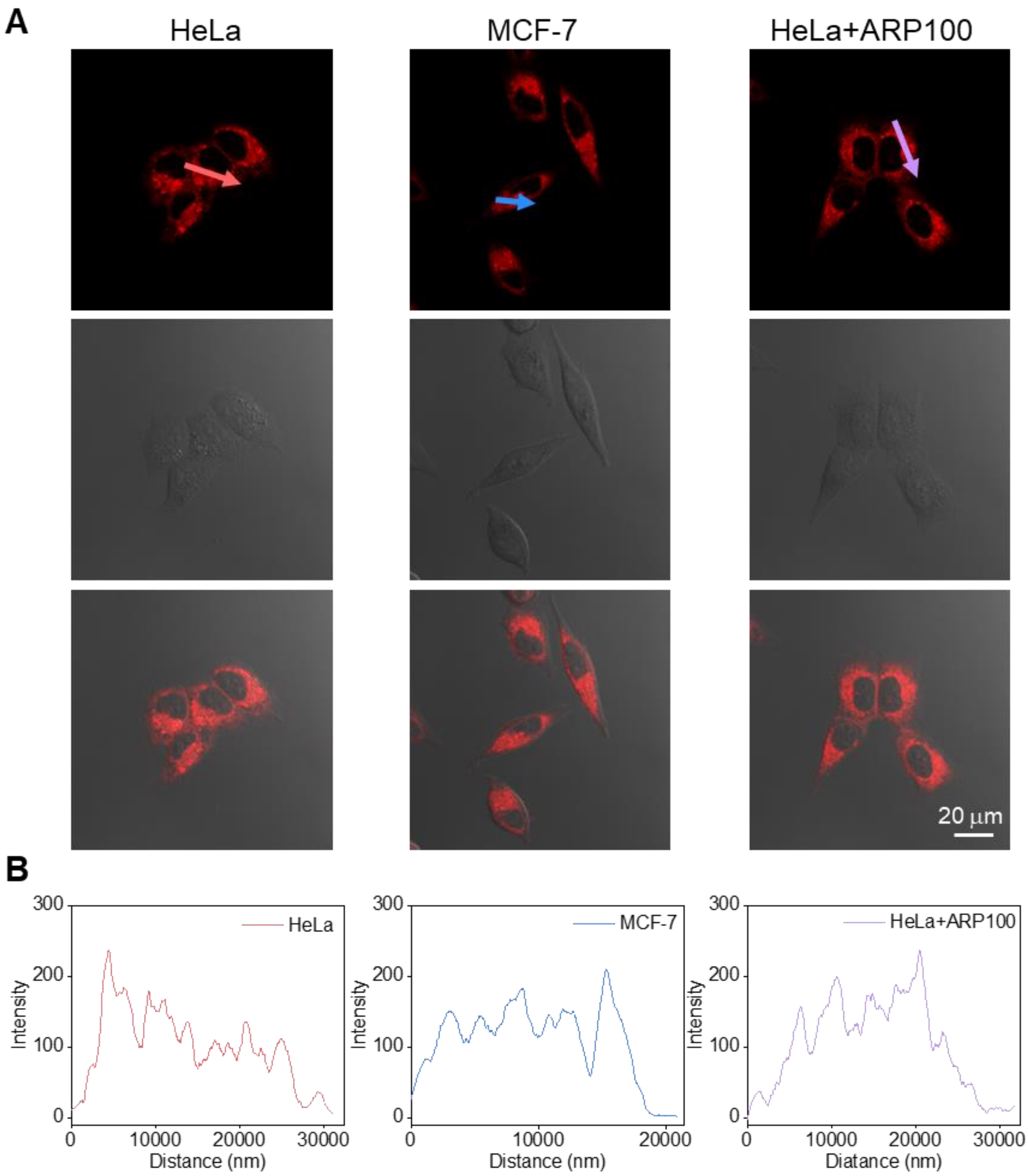

Figure S20. CLSM images of HeLa cells, MCF-7 cells, and HeLa+ARP100 incubated with PyTPA. A) CLSM images and B) intensity of HeLa cells, MCF-7 cells, and HeLa+ARP100 incubated with PyTPA (20 μM) for 4 h. The linear region across the HeLa cells, MCF-7 cells, and HeLa cells+ARP100 were marked by red arrows, blue arrows, and purple arrows respectively. A 488 nm laser was chosen for the excitation of PyTPA, the emission was collected at 600-740 nm (red fluorescence channel).

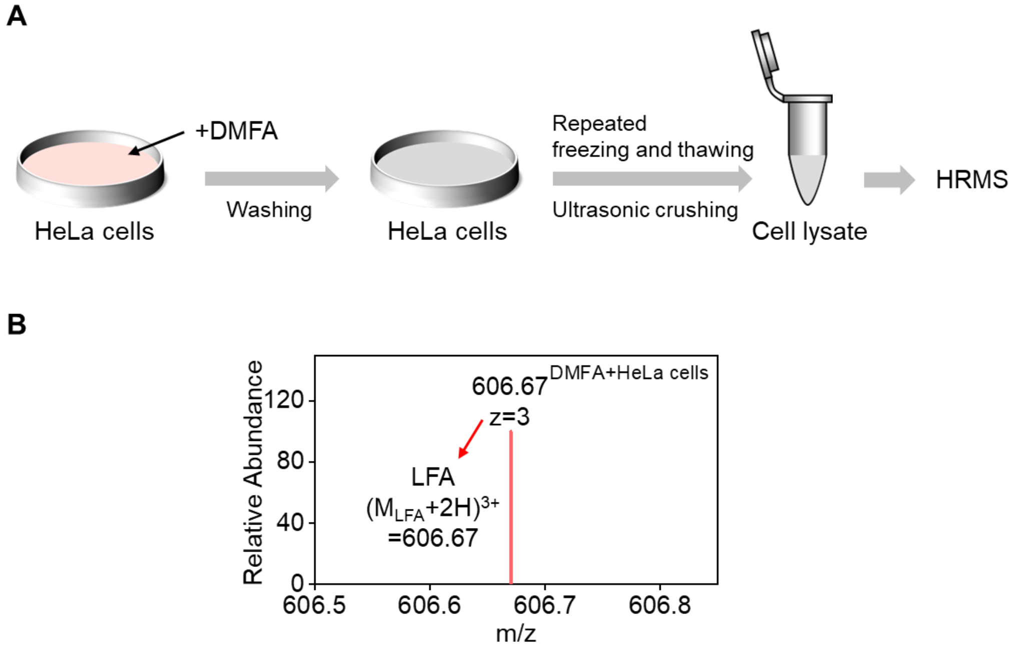

Figure S21. HRMS assay of HeLa cell lysates. A) Schematic diagram and B) HRMS spectrum of HeLa cell lysates.

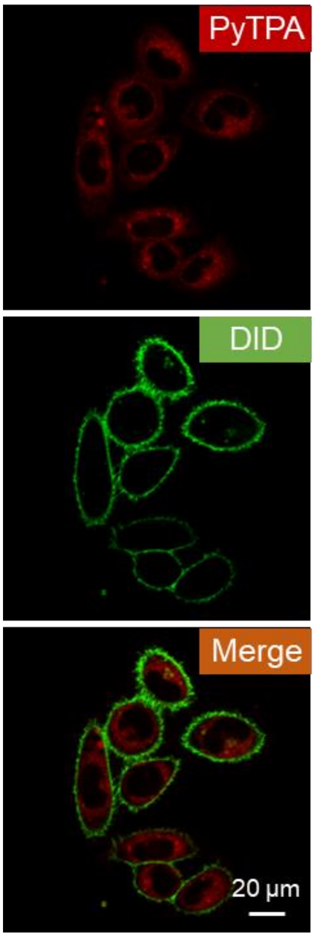

Figure S22. CLSM images of HeLa cells incubated with PyTPA (20 μM) for 4 h and then incubated with DID for 15 min. A 488 nm laser was chosen for the excitation of PyTPA, the emission was collected at 600-740 nm (red fluorescence channel). A 633 nm laser was chosen for the excitation of DID, the emission was collected at 645-680 nm (green fluorescence channel).

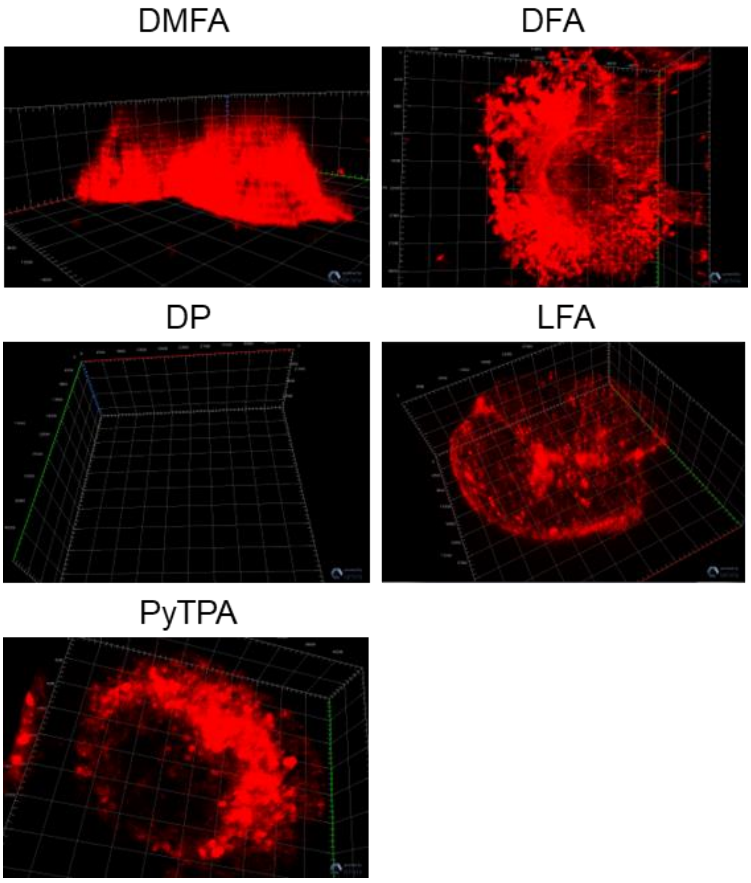

Figure S23. 3D CLSM images of HeLa cells after incubation with different probes. 3D CLSM images of HeLa cells after incubation with DMFA, DFA, DP, LFA, and PyTPA for 4 h. The concentrations of DMFA, DFA, DP, LFA, and PyTPA were 20 μM. The red fluorescence signal (AIEgen) of DMFA and LFA were on the cell membranes, and the red fluorescence signal (AIEgen) of DFA and PyTPA were inside the cells. A 488 nm laser was chosen for the excitation of the probes, the emission was collected at 600-740 nm (red fluorescence channel).

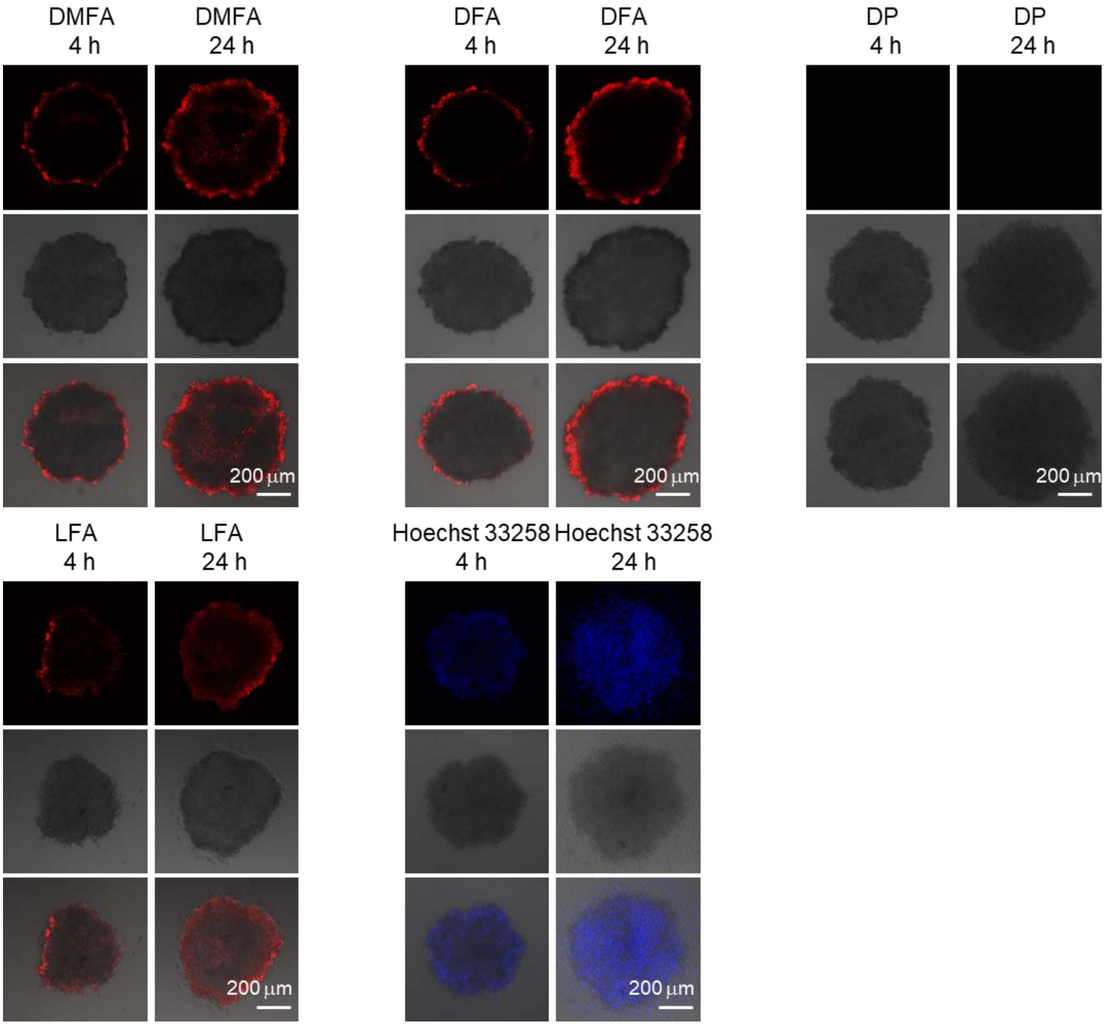

Figure S24. CLSM images of MCTs incubated with different probes. CLSM images of MCTs incubated with DMFA, DFA, DP, LFA, and Hoechst 33258 for 4 h and 24 h. The concentrations of DMFA, DFA, DP, LFA, and Hoechst 33258 were 20 μM. A 488 nm laser was chosen for the excitation of DMFA, DFA, DP, and LFA, the emission was collected at 600-740 nm (red fluorescence channel). A 405 nm laser was chosen for the excitation of Hoechst 33258, the emission was collected at 410-460 nm (blue fluorescence channel).

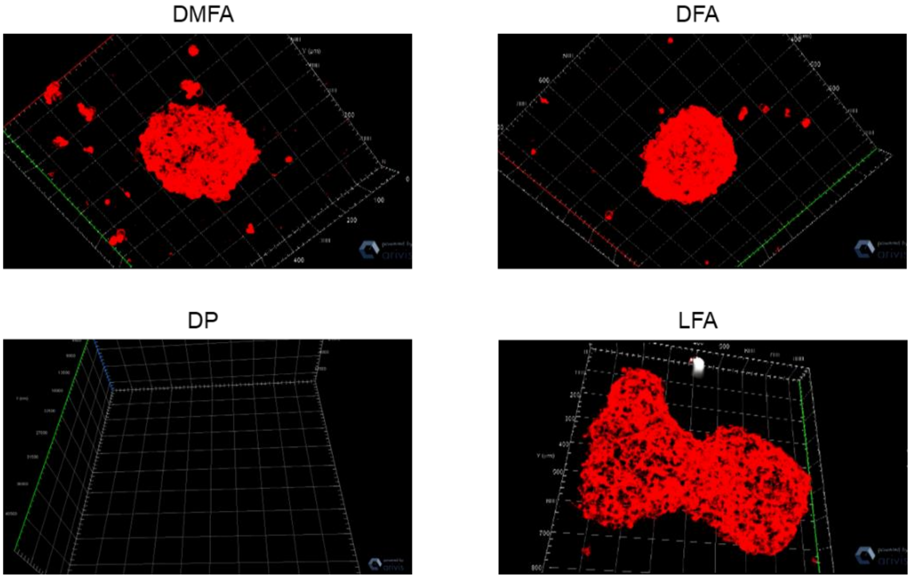

Figure S25. 3D CLSM images of MCTs incubated with different probes. 3D CLSM images of MCTs incubated with DMFA, DFA, DP, and LFA for 4 h. The concentrations of DMFA, DFA, DP, and LFA were 20 μM. A 488 nm laser was chosen for the excitation of DMFA, DFA, DP, and LFA, the emission was collected at 600-740 nm (red fluorescence channel).

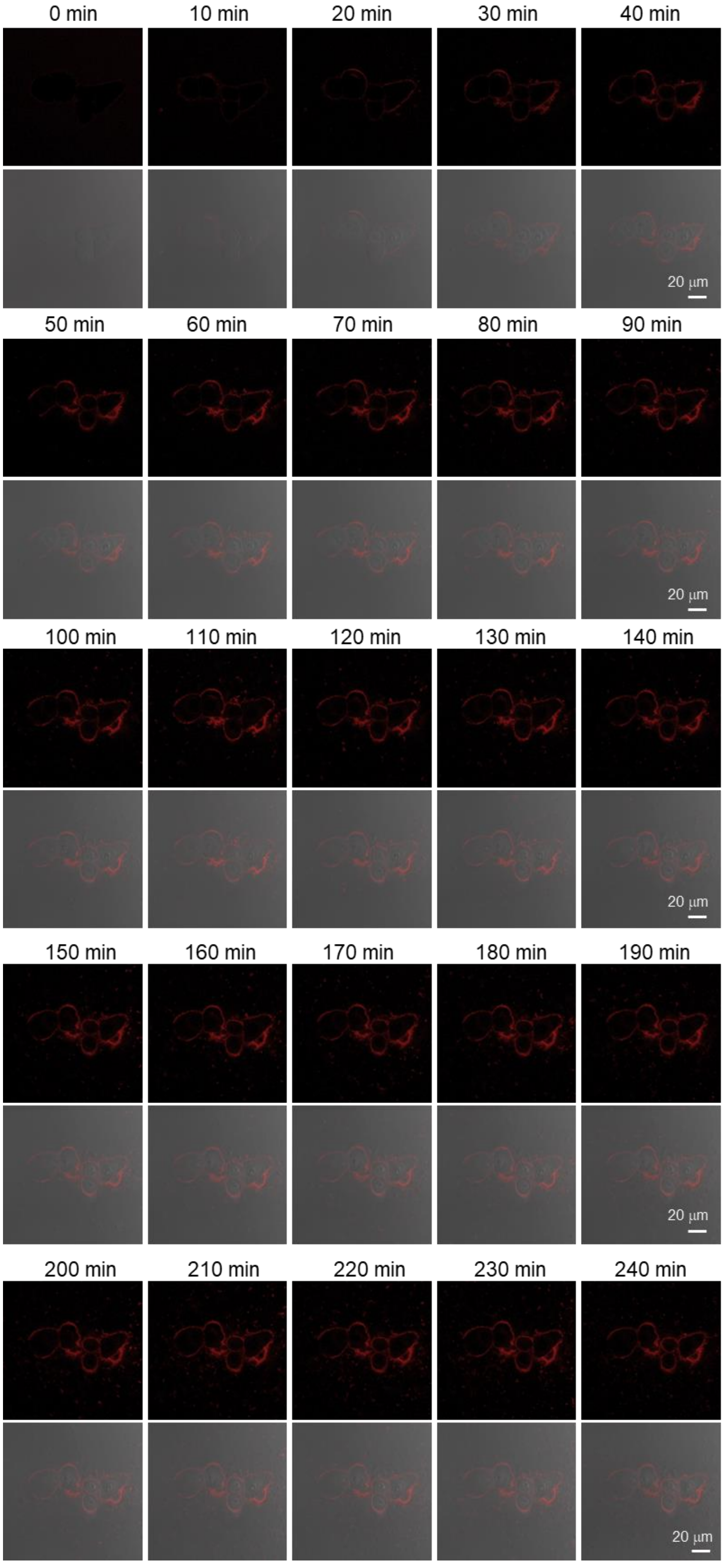

Figure S26. Real-time CLSM images of HeLa cells incubated with DMFA. Real-time CLSM images of HeLa cells incubated with DMFA (20 μM) for 4 h. A 488 nm laser was chosen for the excitation of DMFA, the emission was collected at 600-740 nm (red fluorescence channel).

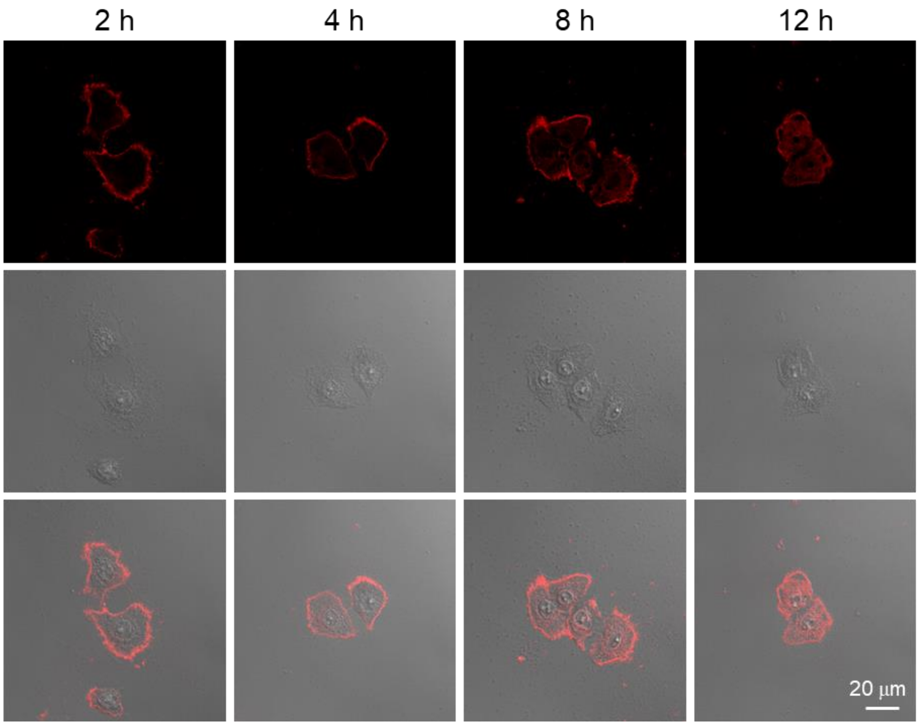

Figure S27. CLSM images of HeLa cells incubated with DFA for different times. CLSM images of HeLa cells incubated with DFA (20 μM) for 2 h, 4 h, 8 h and 12 h. A 488 nm laser was chosen for the excitation of DFA, the emission was collected at 600-740 nm (red fluorescence channel).

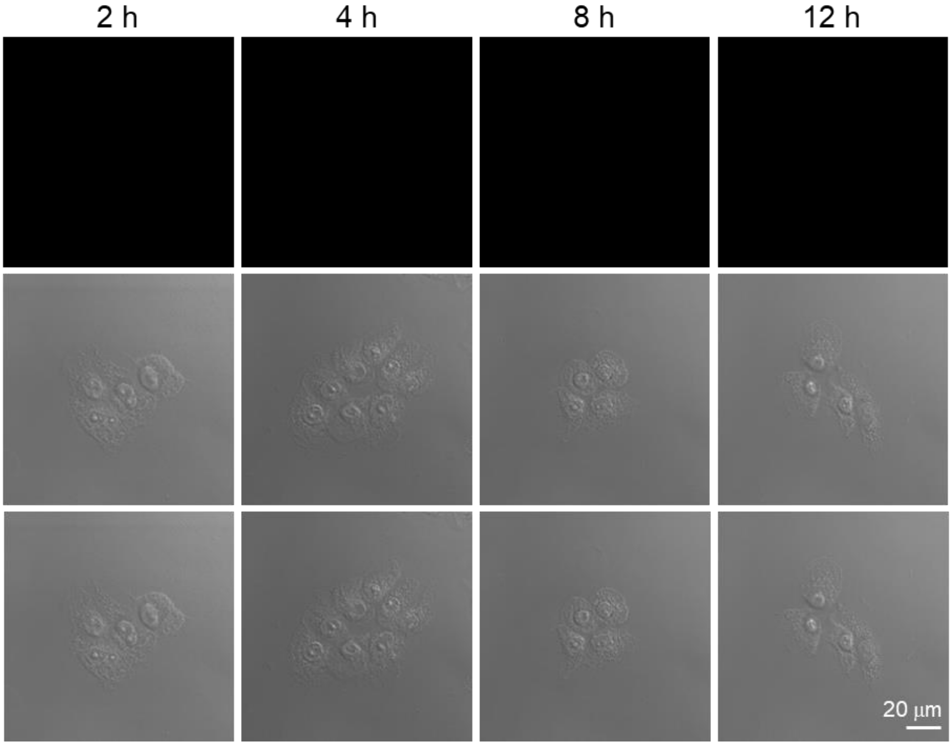

Figure S28. CLSM images of HeLa cells incubated with DP for different times. CLSM images of HeLa cells incubated with DP (20 μM) for 2 h, 4 h, 8 h and 12 h. A 488 nm laser was chosen for the excitation of DP, the emission was collected at 600-740 nm (red fluorescence channel).

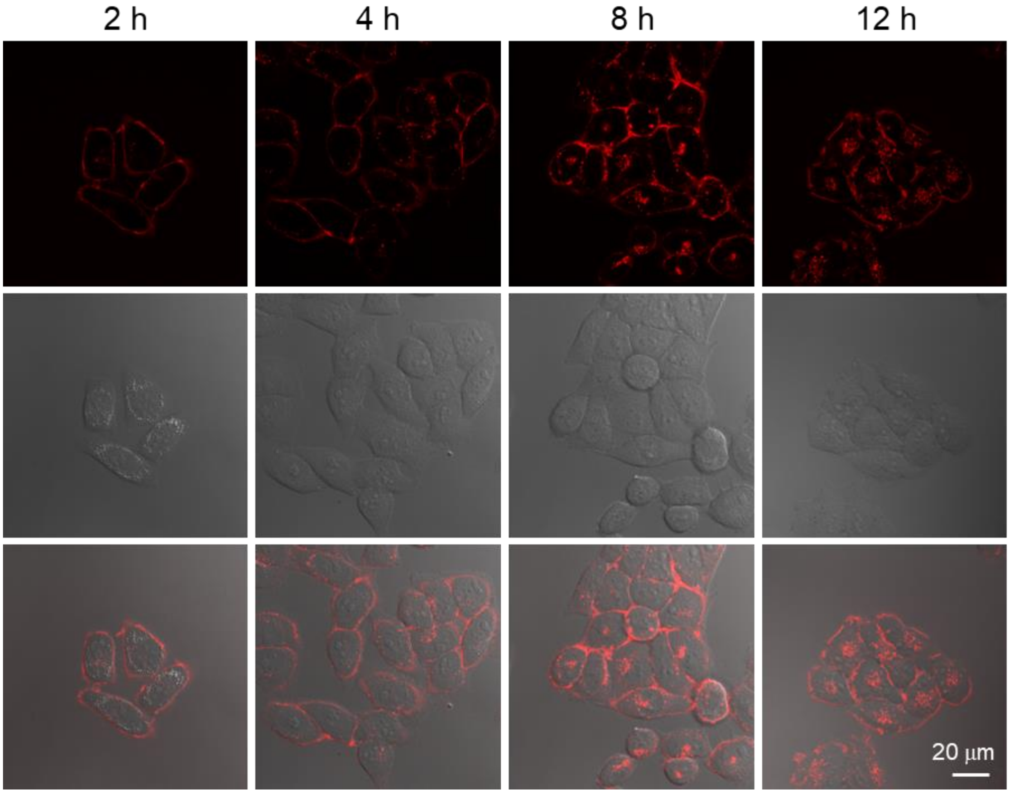

Figure S29. CLSM images of HeLa cells incubated with LFA for different times. CLSM images of HeLa cells incubated with LFA (20 μM) for 2 h, 4 h, 8 h and 12 h. A 488 nm laser was chosen for the excitation of LFA, the emission was collected at 600-740 nm (red fluorescence channel).

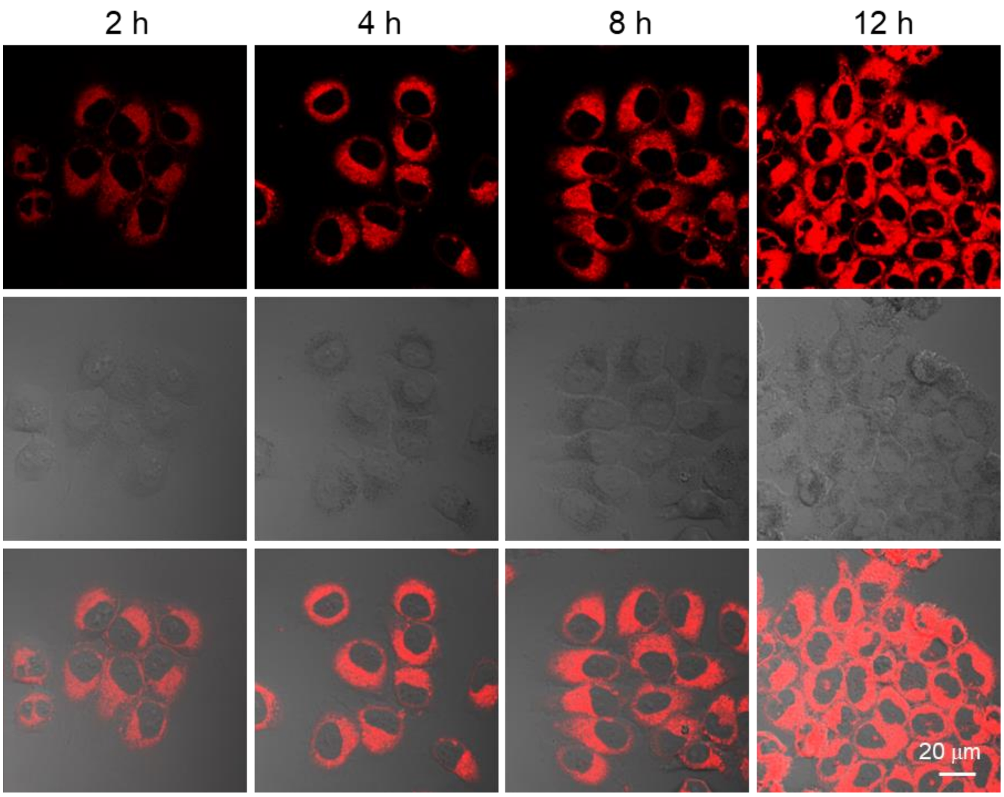

Figure S30. CLSM images of HeLa cells incubated with PyTPA for different times. CLSM images of HeLa cells incubated with PyTPA (20 μM) for 2 h, 4 h, 8 h and 12 h. A 488 nm laser was chosen for the excitation of PyTPA, the emission was collected at 600-740 nm (red fluorescence channel).

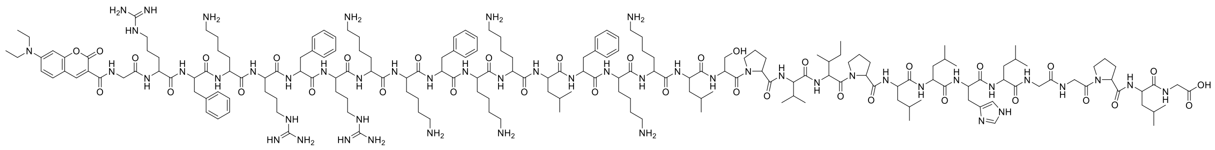

Figure S31. Chemical structure of DP-DEAC (DP labeled with DEAC fluorescent molecules).

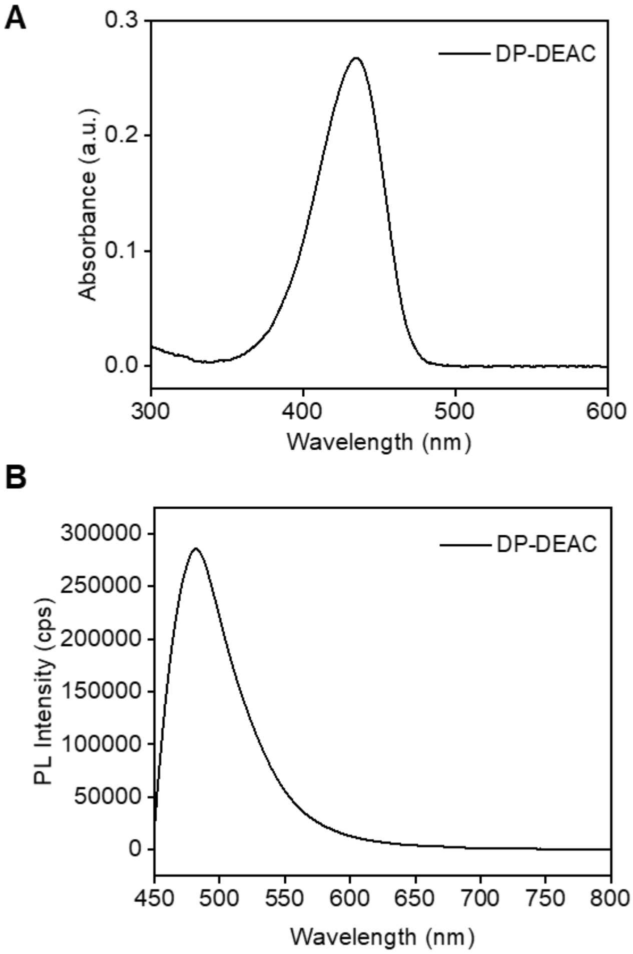

Figure S32. UV-vis assay and flourescence assay. A) UV-vis spectra and B) fluorescence spectra of DP-DEAC (10 μM).

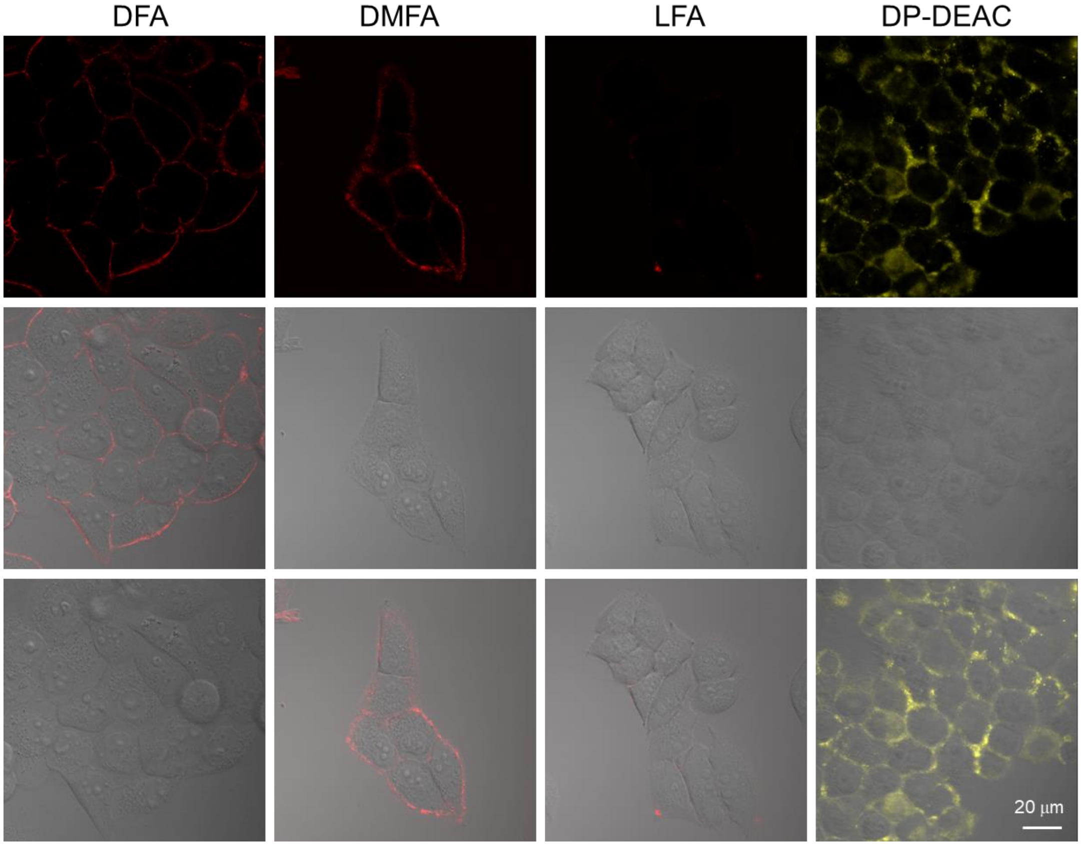

Figure S33. CLSM images of HeLa cells incubated with different probes. CLSM images of HeLa cells incubated with DFA, DMFA, LFA, and DP-DEAC for 5 min. The concentrations of DFA, DMFA, LFA, and DP-DEAC were 10 μM. A 488 nm laser was chosen for the excitation of DFA, DMFA and LFA, the emission was collected at 600-740 nm (red fluorescence channel). A 405 nm laser was chosen for the excitation of DP-DEAC, the emission was collected at 450-530 nm (yellow fluorescence channel).

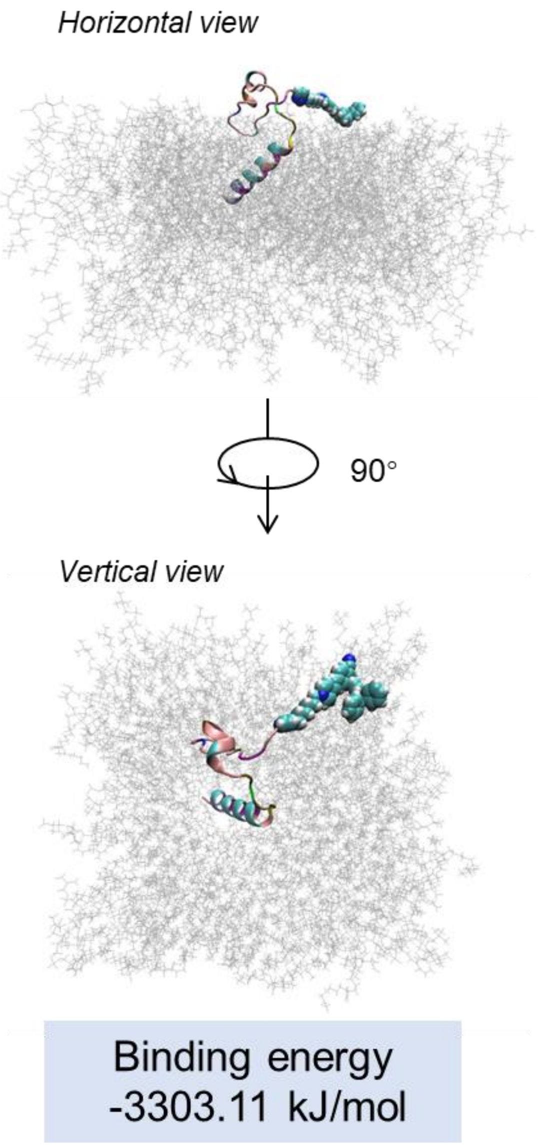

Figure S34. Computational simulation of the binding system between DMFA and cell membrane.

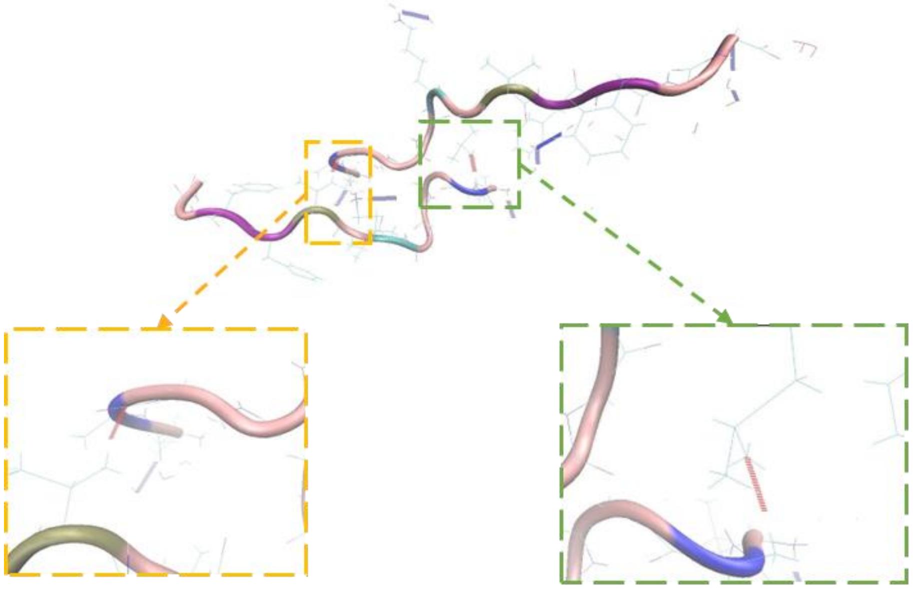

Antiparallel β-sheet structure of LF (binding energy: -75.61 kJ/mol)

Figure S35. Computational simulation of LF structure. The binding energy of LF assembled into LF anti-parallel β-sheet structure and the structural simulation of the simplified model of LF anti-parallel β-sheet structure. The red dotted lines in representative position represented the hydrogen bonds between amino acids, and the blue dotted lines in representative position represented the hydrogen bonds between amino acids and H_2O. The size of the periodic box was 10 nm × 10 nm × 10 nm.

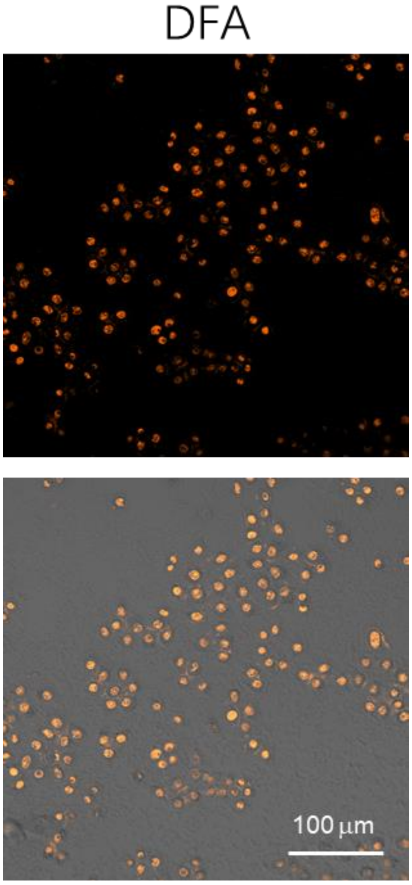

Figure S36. CLSM images of HeLa cells treated with different probes. CLSM images of HeLa cells treated with DFA (20 μM) for 4 h and then incubated with PI for 20 min. A 543 nm laser was chosen for the excitation of PI, the emission was collected at 600-660 nm (orange fluorescence channel).

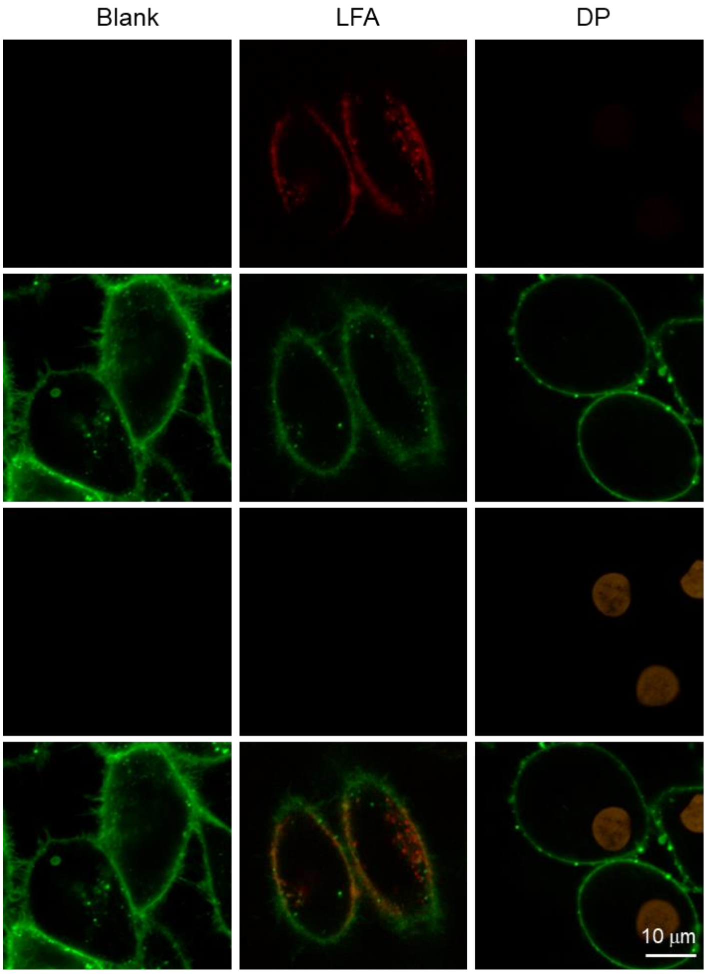

Figure S37. CLSM images of HeLa cells incubated with different probes. CLSM images of HeLa cells incubated with different probes (20 μM, 4 h), PI (20 min), and DID (15 min) in turn. A 488 nm laser was chosen for the excitation of LFA and DP, the emission was collected at 670-740 nm (red fluorescence channel). A 633 nm laser was chosen for the excitation of DID, the emission was collected at 645-680 nm (green fluorescence channel). A 543 nm laser was chosen for the excitation of PI, the emission was collected at 600-660 nm (orange fluorescence channel).

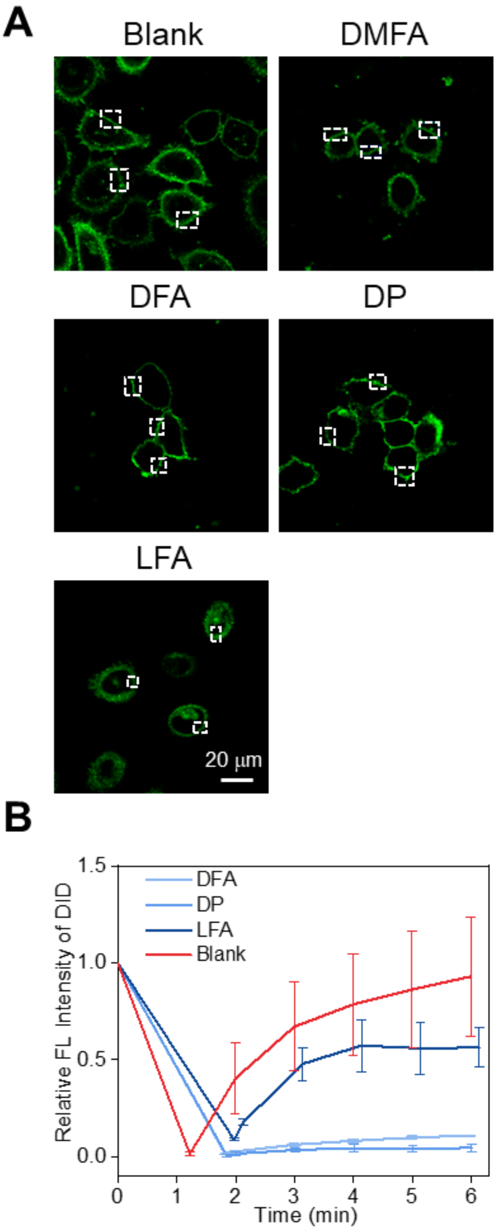

Figure S38. Photobleaching assay. A) CLSM images of HeLa cells treated with or without different probes (20 μM) for 4 h and then incubated with DID (20 μM) for 15 min. The photobleaching area was marked with a white box. A 633 nm laser was chosen for the excitation of DID, the emission was collected at 645-680 nm (green fluorescence channel). B) The fluorescence recovery of DID which was incubated with HeLa cells pretreated with different probes (n=3). Data were presented as mean ± SD.

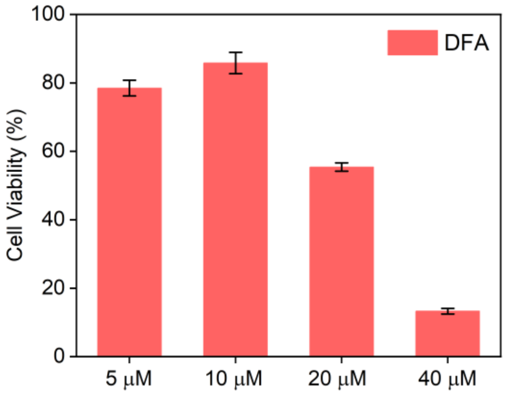

Figure S39. Cell viability of HeLa cells incubated with DFA. Cell viability of HeLa cells incubated with different concentrations of DFA for 24 h (n=3). Data were presented as mean ± SD.

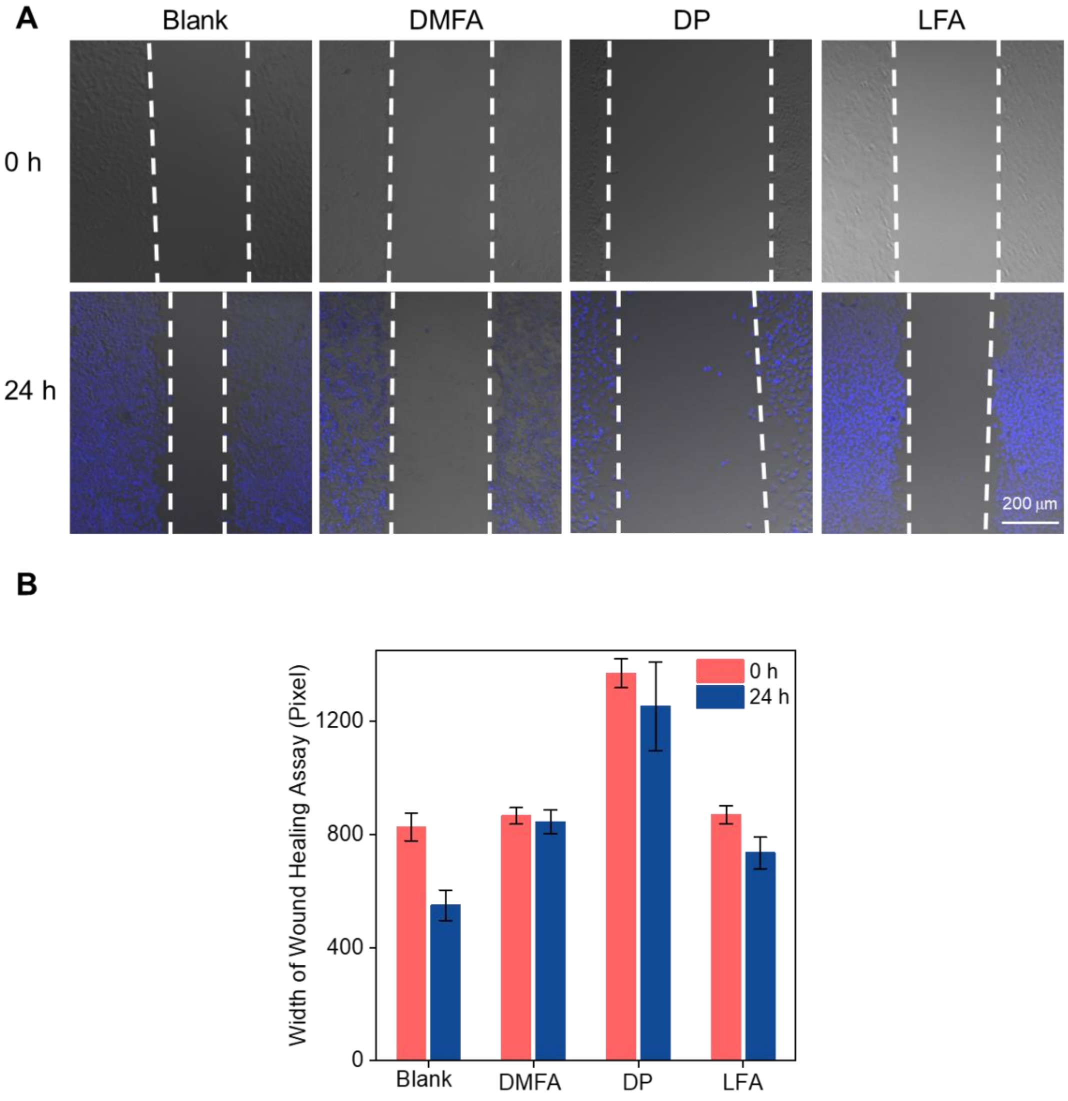

Figure S40. Scratch test. A) CLSM images and B) quantitative analysis of HeLa cells incubated with different probes (20 μM) for 24 h and then stained with Hoechst 33258 for 15 min. A 405 nm laser was chosen for the excitation of Hoechst 33258, the emission was collected at 410-460 nm. Data were presented as mean ± SD.

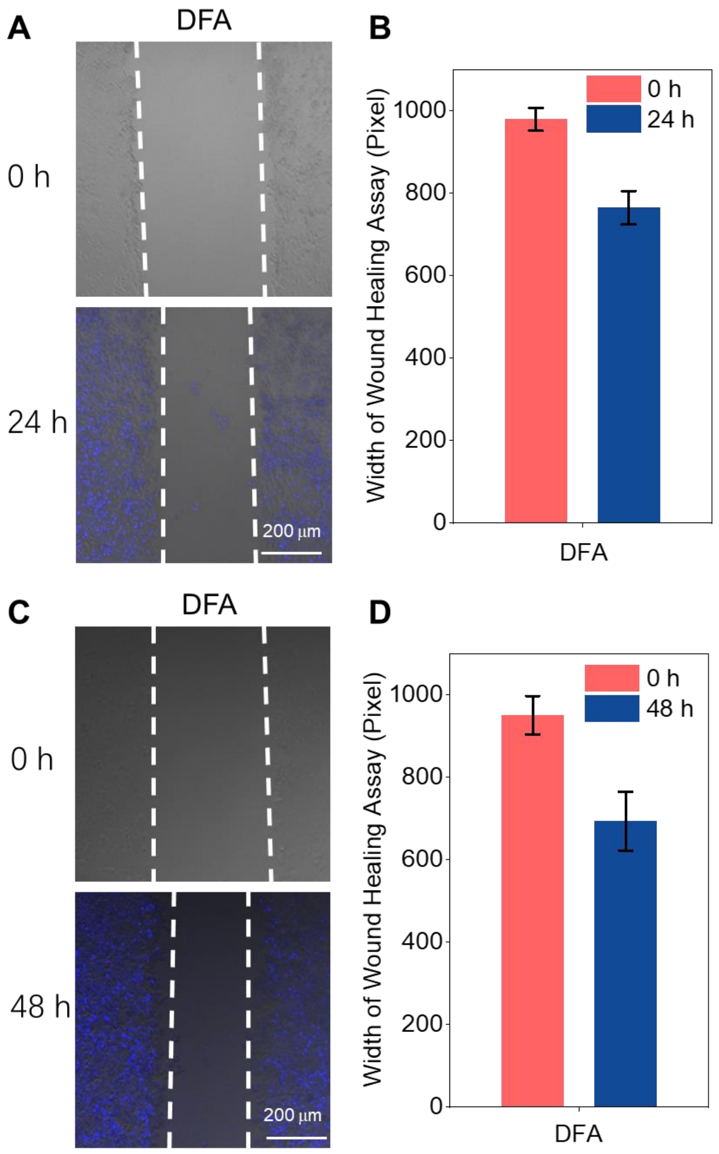

Figure S41. Scratch test of DFA group. A) CLSM images and B) quantitative analysis of HeLa cells incubated with DFA (20 μM) for 24 h and then stained with Hoechst 33258 for 15 min. C) CLSM images and D) quantitative analysis of HeLa cells incubated with DFA (20 μM) for 48 h and then incubated with Hoechst 33258 for 15 min. Data were presented as mean ± SD. A 405 nm laser was chosen for the excitation of Hoechst 33258, the emission was collected at 410-460 nm.

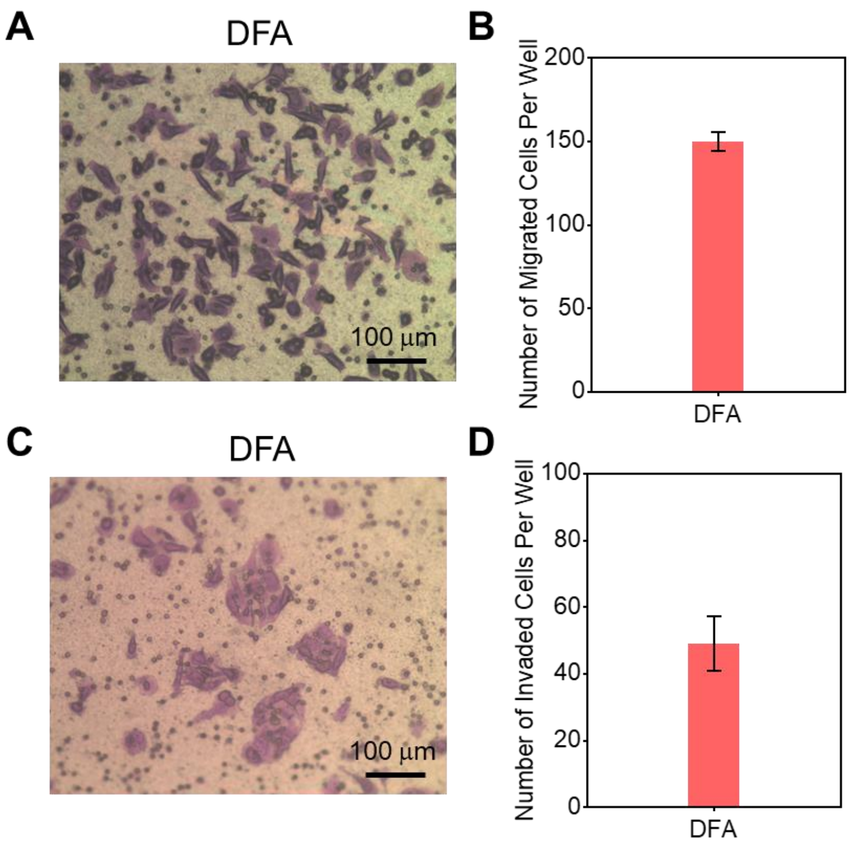

Figure S42. Transwell migration and transwell invasion assay of DFA group (n=3). A) Transwell migration microscopy images and B) quantitative analysis of HeLa cells incubated with DFA (20 µM) for 24 h. C) Transwell invasion microscopy images and D) quantitative analysis of HeLa cells incubated with DFA (20 µM) for 24 h. Data were presented as mean ± SD.

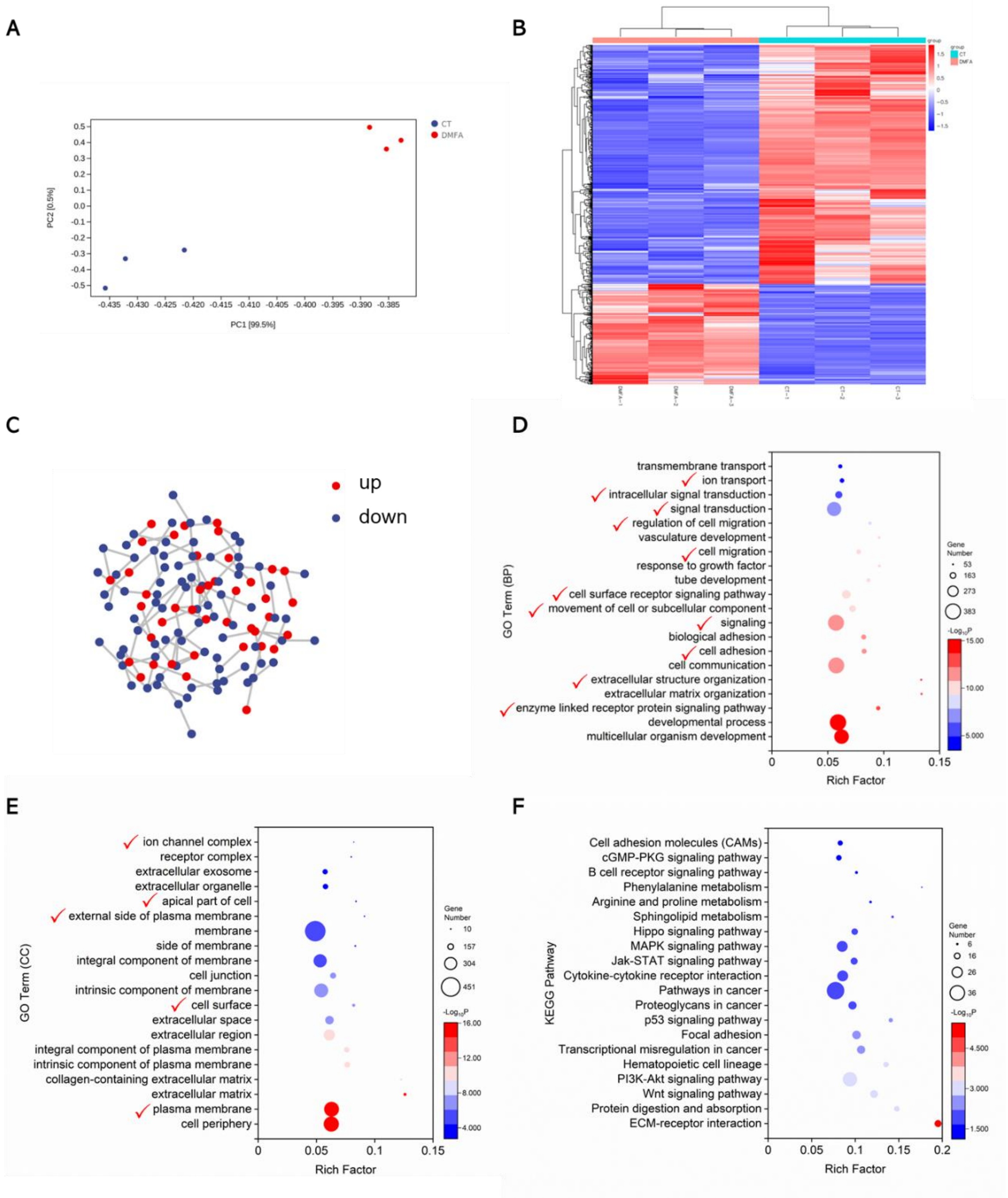

Figure S43. Transcriptome test. A) PCA analysis, B) Heat map, and C) Protein network differentially expressed genes between HeLa cells treated with or without DMFA (20 μM) for 24 h. D) GO BP term enrichment analysis, E) GO CC term enrichment analysis, and F) KEGG pathway enrichment analysis of differentially expressed genes between HeLa cells treated with or without DMFA (20 μM) for 24 h.

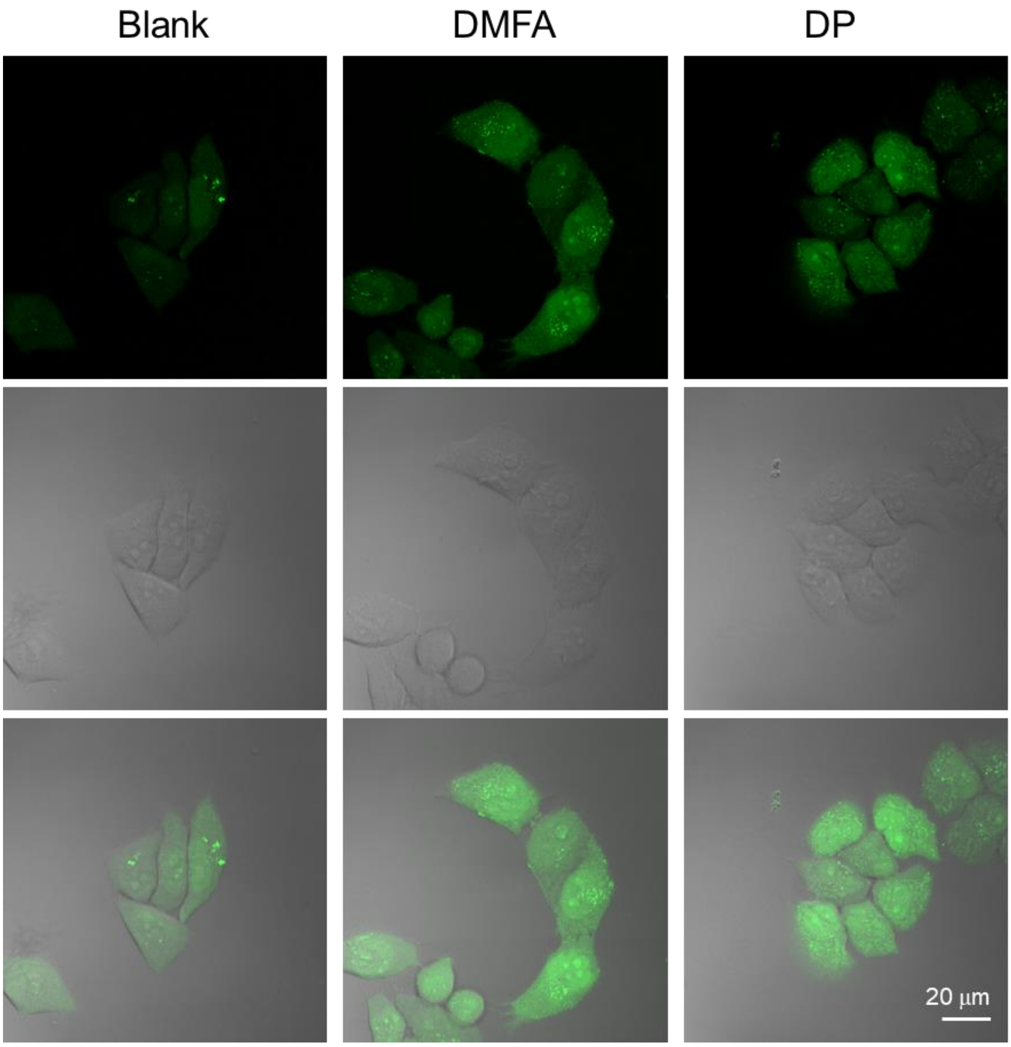

Figure S44. CLSM images of HeLa cells incubated with different probes. CLSM images of HeLa cells incubated with DMFA and DP (20 μM) for 30 min, and then incubated with Fura-2 AM (1 μM) for 30 min. A 405 nm laser was chosen for the excitation of Fura-2 AM, the emission was collected at 490-520 nm (green fluorescence channel).

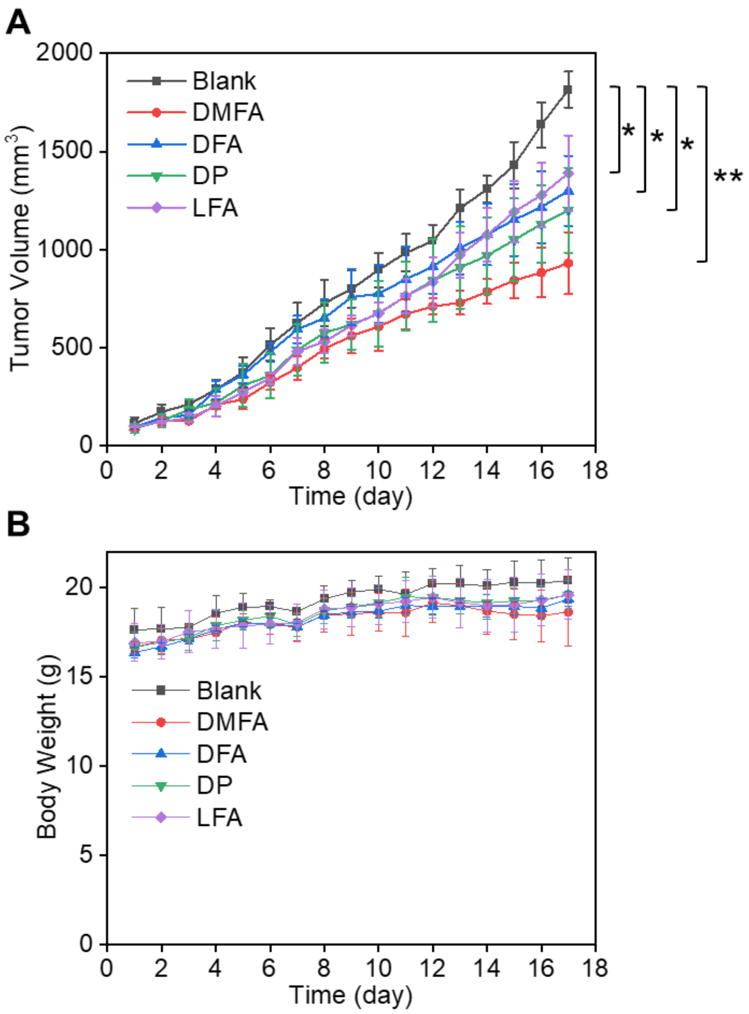

Figure S45. Tumor volume change of tumors and body weight change of tumor bearing mice (n=3 per group). A) Tumor volume change of tumors in each group during treatment. B) Body weight change of tumor bearing mice in each group during treatment. Data were expressed as mean ± SD. **p*<0.05, ***p*<0.01.

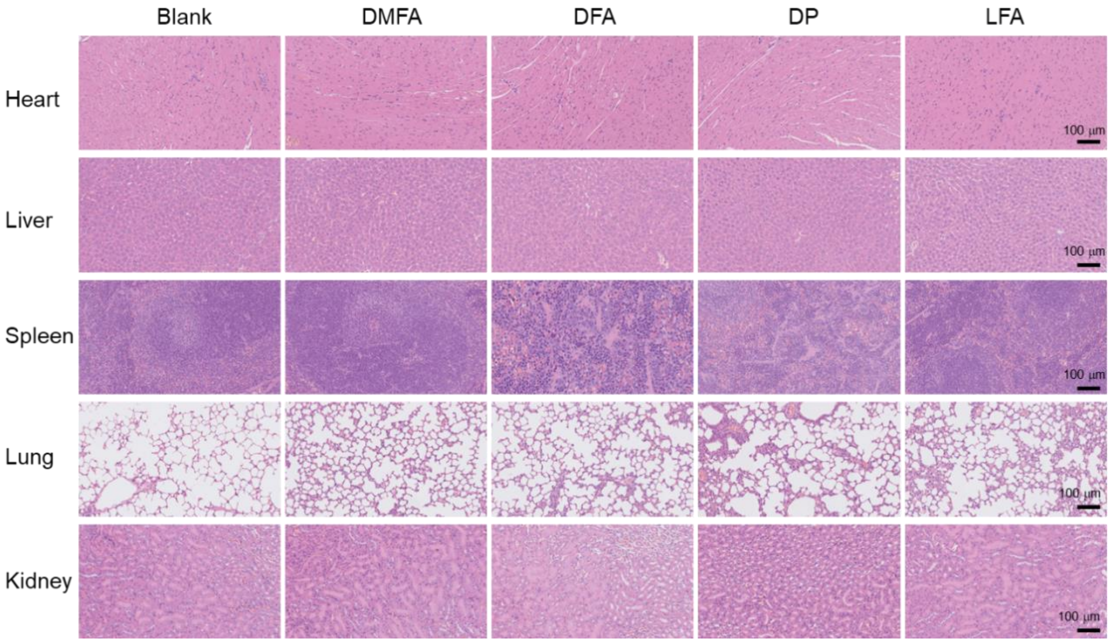

Figure S46. H&E-staining tissue. H&E-staining tissue sections from heart, liver, spleen, lung, and kidney of HeLa tumor-bearing mice with different treatments.

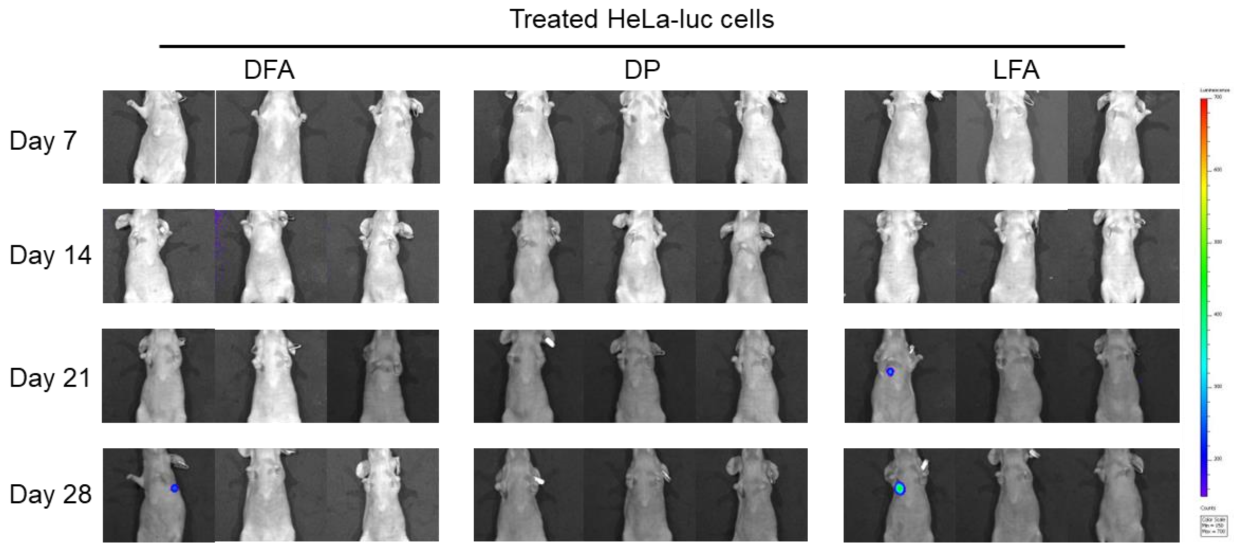

Figure S47. *In vivo* bioluminescence imaging of nude mice after different treatments (n=3 per group).

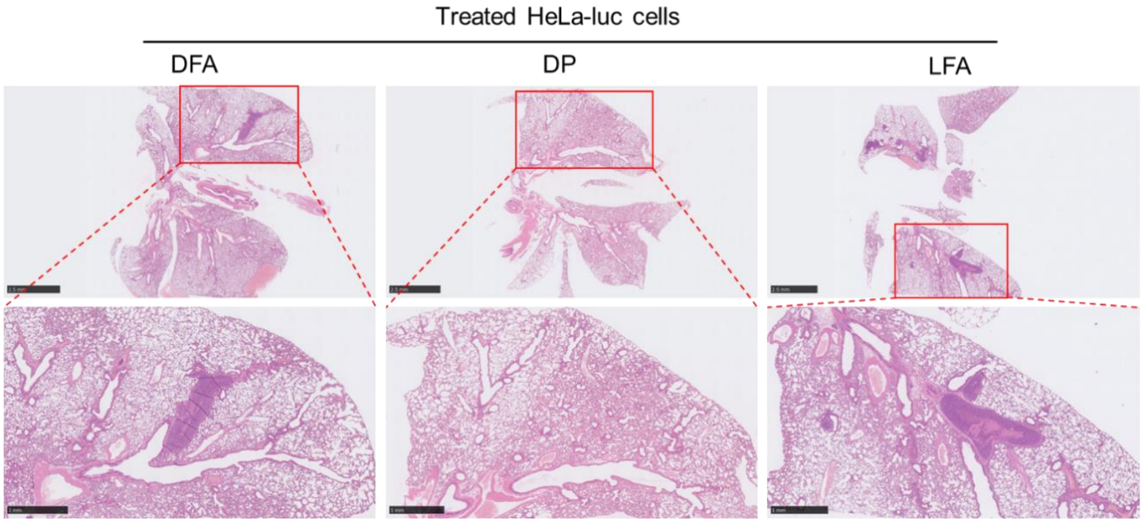

Figure S48. H&E-stained tissue sections from lung of nude mice. H&E-stained tissue sections from lung of nude mice after received different treatments. The scale bars of the first line of images were 2.5 mm, and the scale bars of the enlarged images were 1 mm.
